# Accurate prediction of dynamic protein–ligand binding using *P‐score* ranking

**DOI:** 10.1002/jcc.27370

**Published:** 2024-04-22

**Authors:** Peter E. G. F. Ibrahim, Fabio Zuccotto, Ulrich Zachariae, Ian Gilbert, Mike Bodkin

**Affiliations:** ^1^ Drug Discovery Unit, Division of Biological Chemistry and Drug Discovery University of Dundee Dundee UK

**Keywords:** binding pose prediction, dynamic average quantum mechanics fragment molecular orbital, *P‐score*, supervised molecular dynamics

## Abstract

Protein–ligand binding prediction typically relies on docking methodologies and associated scoring functions to propose the binding mode of a ligand in a biological target. Significant challenges are associated with this approach, including the flexibility of the protein–ligand system, solvent‐mediated interactions, and associated entropy changes. In addition, scoring functions are only weakly accurate due to the short time required for calculating enthalpic and entropic binding interactions. The workflow described here attempts to address these limitations by combining supervised molecular dynamics with dynamical averaging quantum mechanics fragment molecular orbital. This combination significantly increased the ability to predict the experimental binding structure of protein–ligand complexes independent from the starting position of the ligands or the binding site conformation. We found that the predictive power could be enhanced by combining the residence time and interaction energies as descriptors in a novel scoring function named the *P‐score*. This is illustrated using six different protein–ligand targets as case studies.

## Graphical abstract

The *P‐score* defines a protein–ligand bound pose is a result of dynamic induced‐fit binding event. Therefore, by combing a ligand protein residence time calculated from molecular dynamics with an interaction energy derived from quantum mechanics a novel scoring function, termed the *P‐score*, can accurately predict ligand binding for protein–ligand complexes.

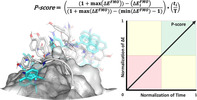

## 1 INTRODUCTION

In the absence of any crystallographic data, the accurate prediction of a ligand's binding mode can be critical for success in structure‐based drug discovery.^1^ Likewise, structure based virtual screening methods, which are widely used for the identification of chemical starting points, rely heavily on accurate pose prediction and scoring.^2^, ^3^, ^4^, ^5^

There is widespread application of protein–ligand docking algorithms in molecular design, yet they possess significant drawbacks. To enable docking at speed a variety of approximations are made to derive a scoring function which albeit renders the scores almost qualitative. The scoring system rarely accounts for the conformational flexibility of either the protein or the ligand associated with induced‐fit or conformational‐selection binding events. Consequently, water and or ion solvation together with the entopic changes associated on ligand binding are poorly handled. Hence, small changes in the binding site conformation or entropic solvation can lead to drastically different docking results.^6^, ^7^, ^8^, ^9^, ^10^, ^11^, ^12^

To address these limitations, molecular mechanics with Poisson‐Boltzmann and surface area (MM‐PBSA) or molecular mechanics with generalized Born and surface area (MM‐GBSA) approaches were introduced to estimate the binding energies, combining molecular mechanics with estimates of solvation energies. These methods were introduced in the late 1990s by the Kollman and Case labs, aiming to estimate the binding free energies, or relative binding free energies, of related compounds and protein–ligand binding modes.^13^, ^14^

Despite their potential, MM‐PBSA/GBSA have several limitations which include the need for prior knowledge of a protein–ligand bound complex to serve as a starting point, although starting conformations can be taken from prior docked poses. Their accuracy for binding poses prediction and ranking the order of compounds according to their affinities is very system dependent.^15^, ^16^

Recent developments in machine and deep learning approaches have led to a possible paradigm shift in the way protein ligand docking solutions are generated. Programs such as LiGANN, DiffDock, or Pocket2Mol generate both molecule and pose simultaneously.^17^, ^18^, ^19^ However, tools such as PoseBusters or PoseCheck which were developed to assess the geometric and interaction quality of generated ligands have highlighted there is significant room for improvement compared to solutions derived from physics‐based approaches.^20^, ^21^

**FIGURE 1:**
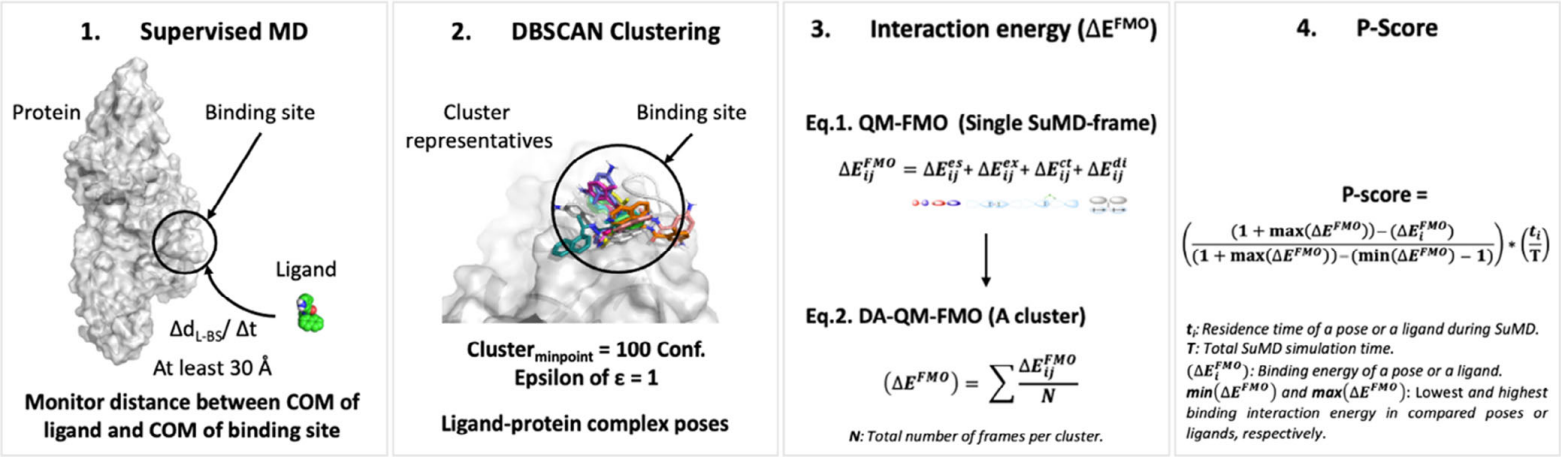
Schematic diagram of the binding pose prediction and scoring, the *P‐score* (SuMD X DA‐QM‐FMO → *P‐score*). Δ*E*, interaction energy; COM. centre of mass; *t*, residence time.

To overcome the issues outlined above, we introduce a novel protocol (Figure 1), which combines supervised molecular dynamics (SuMD) with dynamical averaging of quantum mechanics fragment molecular orbital (DA‐QM‐FMO). SuMD allows a dynamical sampling of a protein–ligand system in a fully solvated environment with protein flexibility and ligand conformational sampling. DA‐QM‐FMO estimates the average potential binding interaction energies from dynamically generated protein–ligands binding modes. In this study, DA‐QM‐FMO was able to outperform simple docking or MM‐PBSA/GBSA at predicting the experimental binding pose.

We believe limitations due to the length of the SuMD simulations and the approximations made in energy calculations techniques, accounts for the fact that these calculations on their own did not fully replicate the experimental data. However, by combining the residence time and binding interaction energy the method was able to improve the predictive power. We refer to the combination of descriptors as the *P‐score* (Figure 1;4). The *P‐score* prioritizes protein–ligand binding poses based on stability and interaction energy of binding modes. We apply the *P‐score* theory using six different case studies, with published crystal structures from the Protein Data Bank (PDB); Papain‐like protease protein (PL‐pro) and Main‐protease (Mpro) for SARS‐CoV‐2, Heat shock protein 90 (Hsp90), p38 Kinase (p38), Myeloid Cell Leukemia 1 (Mcl‐1), and *Pseudomonas*. *aeruginosa* LpxC (LpxC).

## 2 MATERIALS AND METHODS

### 2.1 Protein–ligand sampling phase

SuMD was used to allow a whole ensemble sampling phase between the ligand and its intended target independent form the initial ligand position either in solvent or within the binding site vicinity. SuMD is a novel molecular dynamics approach developed by Moro et al.^22^, ^23^ that enables the investigation of the molecular recognition pathway between ligand and receptor. SuMD overcomes the conventional constraints associated with the docking methods in protein–ligand binding pose generation.^24^, ^25^

#### 2.1.1 System preparation

Protein structures for five systems; Pl‐pro, Mpro, p38, Hsp90, and Mcl‐1, were prepared with the protein preparation wizard as implemented in Maestro.^26^ Hydrogen atoms were added to the complex and missing atoms in protein side chains were built according to the AMBER16 force field topology.^27^ The N‐ and C‐terminus were capped. LpxC system was built using Metal Center Parameter Builder (MCPB) from Ambertools for the Zn^+2^ binding site using Zinc AMBER force field (ZAFF) for 4‐coordinated zinc metal centers.^28^

#### 2.1.2 Ligand parameterization

All ligands were prepared and treated under physiological conditions of pH = 7.4, throughout the present study. For the MD simulations, the AMBER16 force field was used. Geometry minimization, and ligand parameters were derived with GAFF, as implemented in Ambertools, by using Antechamber and PARMCHK tools. Partial charges were calculated following the procedure suggested by antechamber.^29^, ^30^

#### 2.1.3 Solvated system setup and equilibration

Protein–ligand complexes were assembled with the tleap tool using AMBER16SB, as the force field for the proteins.^31^ The systems were explicitly solvated by a cubic water box with cell borders placed at least 13 Å away from any protein or ligand atom using TIP3P as the water model.^32^ To neutralize the total charge, Na^+^/Cl^−^ counterions were added to a final salt concentration of 0.15 M. The systems were energy minimized for 1000 steps with the conjugate‐gradient method and then 50,000 steps of NVE (100 ps) ensemble (micro‐canonical ensemble; constant number of particles [N], constant volume [V], constant energy [E]) followed by 1 ns of NPT (isothermal–isobaric ensemble; constant number of particles [N], constant pressure [P], constant temperature [T]) simulation, both using a time step of 2 fs and applying harmonic position constraints on protein and ligand atoms, which were gradually reduced with a scaling factor of 0.1. Pressure was maintained at 1 atm using a Berendsen barostat. The Langevin thermostat was set with a low damping constant of 1 ps–1. Bond lengths involving hydrogen atoms were constrained using the M‐SHAKE algorithm. The MD production runs were conducted in a NVT ensemble. Long‐range Coulomb interactions were treated using the particle mesh Ewald summation method (PME) setting the mesh spacing to 1.0 Å. A nonbonded cutoff distance of 9 Å with a switching distance of 7.5 Å was used.^33^, ^34^, ^35^, ^36^

### 2.2 Protein–ligand binding poses clustering using DBSCAN

After concatenating SuMD trajectories, they were subjected to geometric distinction using DBSCAN. This process helped in identifying clusters of similar conformations in the combined trajectory data. DBSCAN is a density‐based clustering algorithm that allows the most populated ligand‐protein states to be identified and distinguished from the background noise.^37^ Each cluster (protein–ligand binding mode) is composed of a collection of MD snapshots “MD frame.” Binding poses are then evaluated using the energy calculation approaches (MM‐PBSA, QM‐FMO, and DA‐QM‐FMO) to estimate the protein–ligand complex binding energy and interaction energy.

### 2.3 Energy calculation

To evaluate the binding energies from the sampling and clustering phases, we make a comparison between MM‐PBSA, Quantum Mechanics fragment molecular orbital (QM‐FMO) method and Dynamical averaging of QM‐FMO (DA‐QM‐FMO). For the MM‐PBSA, molecular mechanics energies were calculated along with generalized Born and surface area continuum solvation using the MMPBSA.py scripts from the AmberTools22 suite (https://ambermd.org/).^38^ QM‐FMO is used for calculating the binding interaction energies described by the inter‐fragment interaction energy (IFIE) and its pair interaction energy decomposition analysis (PIEDA).^39^, ^40^

Inter‐fragment interaction energy (IFIE) analysis estimates protein–ligand interactions with quantum (electronic) effects using a modest level of computational resources. Furthermore, by means of pair interaction energy decomposition analysis (PIEDA), the FMO interaction energy, Δ*E* ^FMO^, is calculated as the sum of five energy terms: electrostatic (Δ*E* ^ES^), exchange repulsion (Δ*E* ^EX^), dispersion (Δ*E* ^DI^), charge transfer with higher‐order mixed terms (Δ*E* ^CT+mix^), and solvation energy, as shown in Equation (1). This approach offers a detailed estimation of protein–ligand interactions in an implicit water, polarizable continuum model (PCM) that has been of great value in a range of drug discovery projects.

(1) $$∆E^{\mathrm{FMO}}={∆E}_{\mathrm{ij}}^{\mathrm{es}}+{∆E}_{\mathrm{ij}}^{\mathrm{ex}}+{∆E}_{\mathrm{ij}}^{\mathrm{ct}+\mathrm{mix}}+{∆E}_{\mathrm{ij}}^{\mathrm{DI}}+∆E^{\text{Gsol}},$$

Δ*E* ^FMO^; interaction energy of a single snapshot (MD frame).

Dynamical averaging of QM‐FMO allows an estimation of the total average interaction energy (Δ*E* ^FMO^) of the protein–ligand complex along the MD trajectory instead of relying only on the interaction energy Δ*E* ^FMO^ for a single snapshot (cluster representative).^41^

The total average interaction energy (Δ*E* ^FMO^) (DA‐QM‐FMO) of a binding mode is obtained by calculating the average interaction energy Δ*E* ^FMO^ of all snapshots found per cluster divided by the number of snapshots within the cluster (MD frames) as shown in Equation (2).^41^

(2) $$\left(∆E^{\mathrm{FMO}}\right)=\sum \frac{{∆E\;}_{i}^{\mathrm{FMO}}}{N},$$

(Δ*E* ^FMO^) DA‐QM‐FMO represents the average Δ*E* ^FMO^ per cluster; *i* and *N* denote the indices of the MD snapshots and the total number of MD snapshots per cluster, respectively.

Prior to the DA‐QM‐FMO calculations, all snapshots were treated with a restrained minimization procedure with the OPLS3e force field to alleviate steric clashes, following the protein preparation wizard protocol of Schrödinger's software suite.^42^ The heavy atoms of the protein were permitted to move up to 0.3 Å from their original position in the crystal structure, while the heavy atoms of the ligand and water molecules were restrained in position with a force of 1.0 kcal/mol/Å; hydrogen atoms were left unrestrained. The restraint weight was set to ensure that the binding poses of the ligands did not deviate far from their initial positions.

Protein residues within a radius of 5 Å from the ligand atoms were included in the FMO calculations. The C‐terminal carboxylic acid of the peptide was capped with N‐methylamine, and the N‐terminal position acetylated while maintaining the geometry of the neighboring residues. A given amino acid, along with its side chain, C‐alpha, and backbone NH, and the carbonyl group of the adjacent amino acid, define each FMO fragment. Fragmentation was carried out according to a well‐established fragmentation strategy inspired from Facio,^43^ in a fully automated high‐throughput python code. The calculations were performed at second order Møller–Plesset perturbation theory (FMO2‐MP2/6‐31G*) and density function tight‐binding (FMO2‐DFTB) theory level, using GAMESS implementation.^44^, ^45^

All calculations were performed on a hybrid CPU/GPU cluster. SuMD simulations were carried out with the ACEMD engine, on a GPU cluster equipped with 16 GPUs (2 NVIDIA GTX 1080 per node on 8 GPUs).^46^

### 2.4 *P‐score* theory for protein–ligand binding pose scoring

**FIGURE 2:**
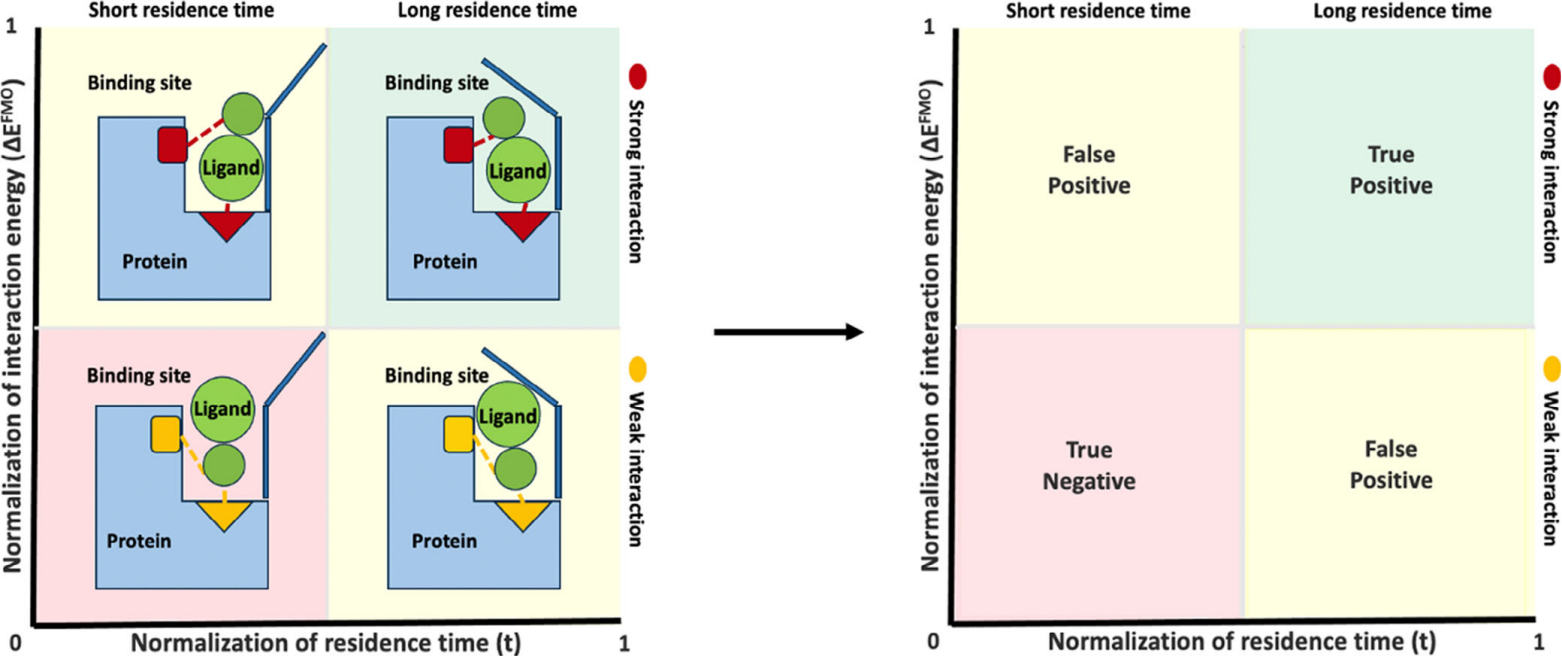
*P‐score* theory diagram, Norm = normalized value of time (*t*) and interaction energy (Δ*E*). Top‐right green box → protein–ligand binding poses or hits of interest. Bottom‐left red box → least interesting protein–ligand binding poses or hits. Top‐left yellow box → protein–ligand binding poses or hits with high affinity but low stability and bottom‐right yellow‐box → protein–ligand binding poses or hits with high stability but low affinity. Vertical line intersecting the x‐axis is the mean of normalized time (*t*) values, and horizontal line intersecting the y‐axis is the mean of normalized values for interaction energy (Δ*E*).

To improve the predictive power for ranking binding poses (cluster representatives), we introduce the *P‐score* as a novel scoring function to eliminate false positives in binding pose ranking. The rationale behind the *P‐score* for protein–ligand binding prediction is that a ligand binding mode of interest is likely to show a lengthened residence time and high degree of affinity in a biological target binding site. In‐silico this is translated into having a prolonged ligand pose stability during the SuMD simulations and a low binding energy or interaction energy (Δ*E*) with binding site residues. Therefore, the normalization of time (*t*) (stability) and Δ*E* (affinity) can be considered as key descriptors for each binding mode or active hit among a virtual screening library. A binding mode can be distinguished using the *P‐score* diagram represented in a quadrant matrix, (Figure 2).

The *P‐score* defines a protein–ligand bound pose is a result of dynamic induced‐fit binding event, that relies on the residence time (*t*) and total average interaction energy (Δ*E* ^FMO^) for each binding mode or hit, using the following Equation (3):

(3) $$P-\text{score}=\left(\frac{\left(1+\max\left(∆E^{\mathrm{FMO}}\right)\right)-\left({∆E}_{i}^{\mathrm{FMO}}\right)}{\left(1+\max\left(∆E^{\mathrm{FMO}}\right)\right)-\left(\min\left(∆E^{\mathrm{FMO}}\right)-1\right)}\right)\times \left(\frac{t_{i}}{T}\right),$$

*P‐score* equation: *t*_*i*_ is the residence time of a pose or a ligand. *T* is the total SuMD simulation time. Δ*E*_*i*_ is the interaction energy of a pose or a ligand. min(Δ*E*) and max(Δ*E*) are the lowest and highest interaction energy, in compared poses or ligands, respectively.

The *P‐score* equation is inspired from the rescaling normalization equation where the range of all descriptors should be normalized so that each descriptor contributes proportionately to a final score.^47^ The *P‐score* gives an evaluation of each binding mode by the normalization of the ensemble of all descriptors; time (*t*) and average interaction energy (Δ*E* ^FMO^), rather than using a single descriptor (residence time [*t*] or interaction energy [Δ*E* ^FMO^]). The *P‐score* gives a score between 0 and 1 which is the normalization range of all descriptors included. A *P‐score* value towards 1 means that this binding mode is highly prioritized as a stable conformation of the protein–ligand complex, with a long residence time (*t*) and a low interaction energy (Δ*E* ^FMO^) (True positive). A pose with a *P‐score* value towards 0 is likely to be the least favorable binding mode for the protein–ligand complex, which can be a result of having the shortest residence time, the highest interaction energy, or both combined, (True negative).

## 3 METHODOLOGY PROTOCOL BENCHMARKING

To benchmark our approach, we perform a comparison between Glide docking, induced‐fit docking and SuMD combined with MMPBSA, SuMD combined with QM‐FMO and SuMD combined with DA‐QM‐FMO.

## 4 DOCKING AND INDUCED‐FIT DOCKING

For our first case study, Pl‐pro, GLIDE and Induced‐fit docking modules of the Maestro 12.6.144 software (Schrodinger, LLC, New York, NY, USA)^9^ were used to perform the molecular docking studies. Ligprep (Schrodinger, LLC)^48^ was employed to optimize the energy of all compounds. The *holo‐closed* P‐loop conformation of Pl‐pro (PDB ID: 7JRN) and the *apo‐opened* P‐loop conformation (PDB ID: 6WUU) were prepared for docking using the ‘protein preparation wizard’ in Maestro (Schrodinger, LLC).^26^ Bond orders and formal charges were added to hetero groups and hydrogens were added to all atoms. Side chains not close to the binding cavity and not participating in salt bridges were neutralized to avoid interference with binding interaction energy, and termini were capped with N‐acetyl (ACE) and N‐methyl amide (NMA) groups. The OPLSe force field was used to refine the structure and optimize the hydrogen bond network. Docking was carried out using the standard precision (SP) mode on the protein structure grid, and the Glide score was used to evaluate the final binding pose of protein–ligand complexes.

For the Induced‐fit docking,^49^ the entire receptor molecule was constrained and minimized with an root‐mean‐square deviation (RMSD) cutoff of 0.18 Å to generate the centroid of the residues, and the box size was automatically generated. Initial Glide docking^26^ was carried out for each ligand, and the side chains were automatically trimmed based on the B‐factor. The receptor and ligand van der Waals scaling were both set to 0.5, and 20 poses were generated. Prime side‐chain prediction and minimization were carried out to refine residues within 5.0 Å of the ligand poses, and side chains were optimized to induce the ligand structure and conformation to fit each pose of the receptor structure. Finally, GLIDE standard precision (SP) redocking was performed on the top‐scoring 20 structures overall and structures within 30.0 kcal/mol of the top‐scoring structures. The Induced‐fit docking (IFD) score was generated for each output pose.

## 5 RESULTS AND DISCUSSION

### 5.1 First case study PL‐pro target system

To test *P‐score* protocol, Papain‐like protease protein (PL‐pro) for SARS‐CoV‐2 coronavirus was used as our first case study.^50^ PL‐pro is expressed during SARS‐CoV‐2 viral replication and strong evidence in support of its function suggests it as a promising target in combatting SARS‐CoV‐2 infection.^51^ Multiple crystal structures of SARS‐Cov‐2 PL‐pro, co‐crystallized with inhibitors are available on the Protein Data bank (Table 1).

**TABLE 1:** Five different PL‐pro inhibitors targeting the catalytic binding site with different potencies, originally identified by high‐quality crystallographic structures were collected from the Protein Data Bank database.

Ligands	Lig‐01	Lig‐02	Lig‐03	Lig‐04	Lig‐05
	(PDB ID: 7JIV)	(PDB ID: 7JRN)	(PDB ID: 7RZC)	(PDB ID: 7SDR)	(PDB ID: 7TZJ)
IC50 (μM)	6.4 ± 0.6	2.3 ± 0.2	0.67 ± 0.14	0.67 ± 0.08	0.7 ± 0.1
2D Structures	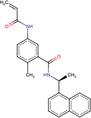	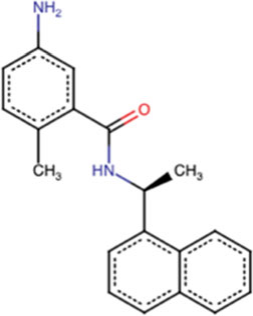	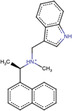	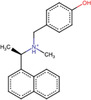	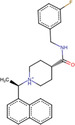

**FIGURE 3:**
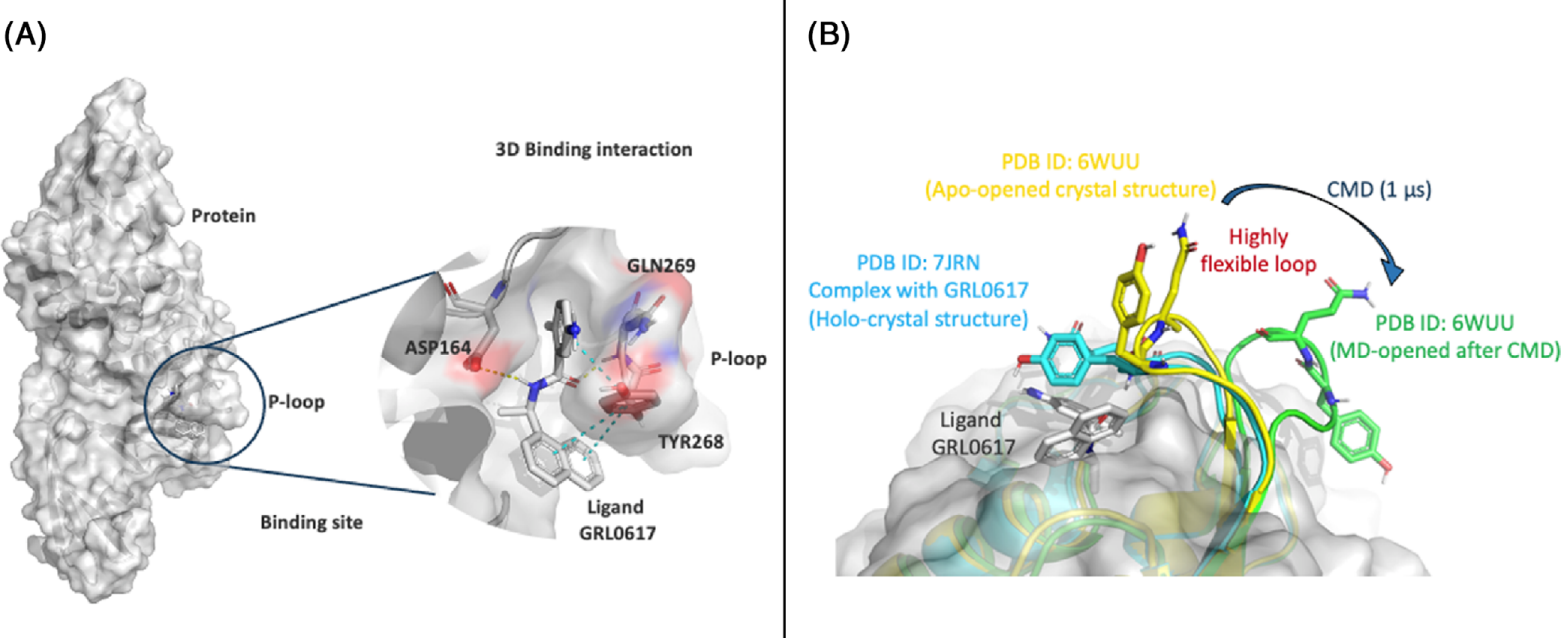
(A). PDB ID: 7JRN in complex with ligand GRL0617. (B) Binding site conformations observed by the P‐loop: *holo‐closed* in ligand–protein crystal complex, *apo‐opened* in apo crystal structure, and CMD‐apo‐opened conformation.

The complexity as well as the flexibility of the Pl‐pro binding pocket, made this test case particularly challenging. The binding site contains of a highly flexible loop named the “P‐loop” that is found in different conformations in the crystal structures. Two crystal structures were chosen for the purpose of including different states of SARS‐CoV‐2 PL‐pro binding site conformation; chain A from the *apo‐form* (PDB ID: 6WUU), and the GRL0617‐bound *Holo‐form* (PDB ID: 7JRN).^52^, ^53^, ^54^ Structural information of the *holo‐form* GRL0617‐bound Pl‐pro (PDB ID: 7JRN), revealed that the catalytic site is formed of a P‐loop composed of TYR‐268 and GLN‐269, forming Pi‐Pi stacking and hydrogen bond interactions with the bound ligand respectively. This conformation of the P‐loop bound to the ligand is named a “*holo‐closed* conformation” (Figure 3A).

In the *apo‐form* of the PL‐pro (PDB ID: 6WUU), the P‐loop showed an open conformation with about 90° difference in orientation of the side chains of the TYR‐268 and GLN‐269. This conformation of the P‐loop is named an “*apo‐opened* conformation” (Figure 3B). This P‐loop acts as a one gate‐keeper structure of PL‐pro inhibitors, having a dynamic motion from opened to closed conformation upon ligand binding. Recent studies by Ismail et al, revealed that upon applying 1 μs of classical molecular dynamics simulation to the PL‐pro *apo‐form*, the P‐loop shows a high degree of flexibility, as shown in (Figure 3B).^55^ This creates a critical challenge in the discovery and optimization of new hit compounds against such highly flexible binding sites.

Since accurate prediction of the correct binding‐poses during virtual screening is essential for the reliability of the screening process. Our protocol was applied to a set of five different PL‐pro inhibitors with a range of binding affinities; as shown in Table 1. These are the ligands from *holo‐*structures of PDB IDs: 7JIV, 7JRN, 7RZC, 7SDR, and 7TZJ, targeting the catalytic binding site of Pl‐pro.^56^, ^57^ The ligands were screened against the three binding site conformations formed by the P‐loop, the *holo‐closed*, *apo‐opened* and CMD‐opened conformations.

The *holo‐closed*, *apo‐opened* and *CMD‐apo‐opened* P‐loop conformations forming the binding site of the PL‐pro were used as starting points for SuMD simulations for the five different PL‐pro inhibitors. Each simulation was 200 ns long with three different replicates to ensure sufficient sampling of ligand in the binding site. The SuMD simulations started with the ligands being placed at random coordinates with a distance between 30 and 60 Å from the binding site.

As the ligands reach the vicinity of 9 Å (cut‐off) with the putative binding site in each replicate, different stabilities occur between the ligand and the binding site, suggesting that the ligands had achieved a different conformational sampling while forming initial binding interactions with the receptor binding site residues (Appendix S1; Videos). Treating each binding site conformation separately; *holo‐closed*, the *apo‐opened* and *CMD‐opened* P‐loop conformations, the three replicates of SuMD simulation were concatenated for each ligand, yielding a total of 600 ns simulation time. Filtration was performed before clustering by removal of all SuMD frames with more than 9 Å distance between the ligand and the catalytic cleft. Specifically, a cluster is a protein–ligand complex (a binding pose) formed of at least 100 similar ligand conformations which differ by no more than 1 Å RMSD. The total number of frames in each cluster yields the total residence time (*t*) for its corresponding protein–ligand binding mode, in which a single SuMD frame is equal to 20 ps of simulation. To calculate the interaction energy between the protein and ligands in the generated binding modes (clusters), snapshots for each cluster were extracted every 2 ns. Subsequently, DA‐QM‐FMO‐based interaction energies (Δ*E* ^FMO^) were calculated for each cluster.

### 5.2 PL‐pro system: *Holo‐closed* P‐loop

**FIGURE 4:**
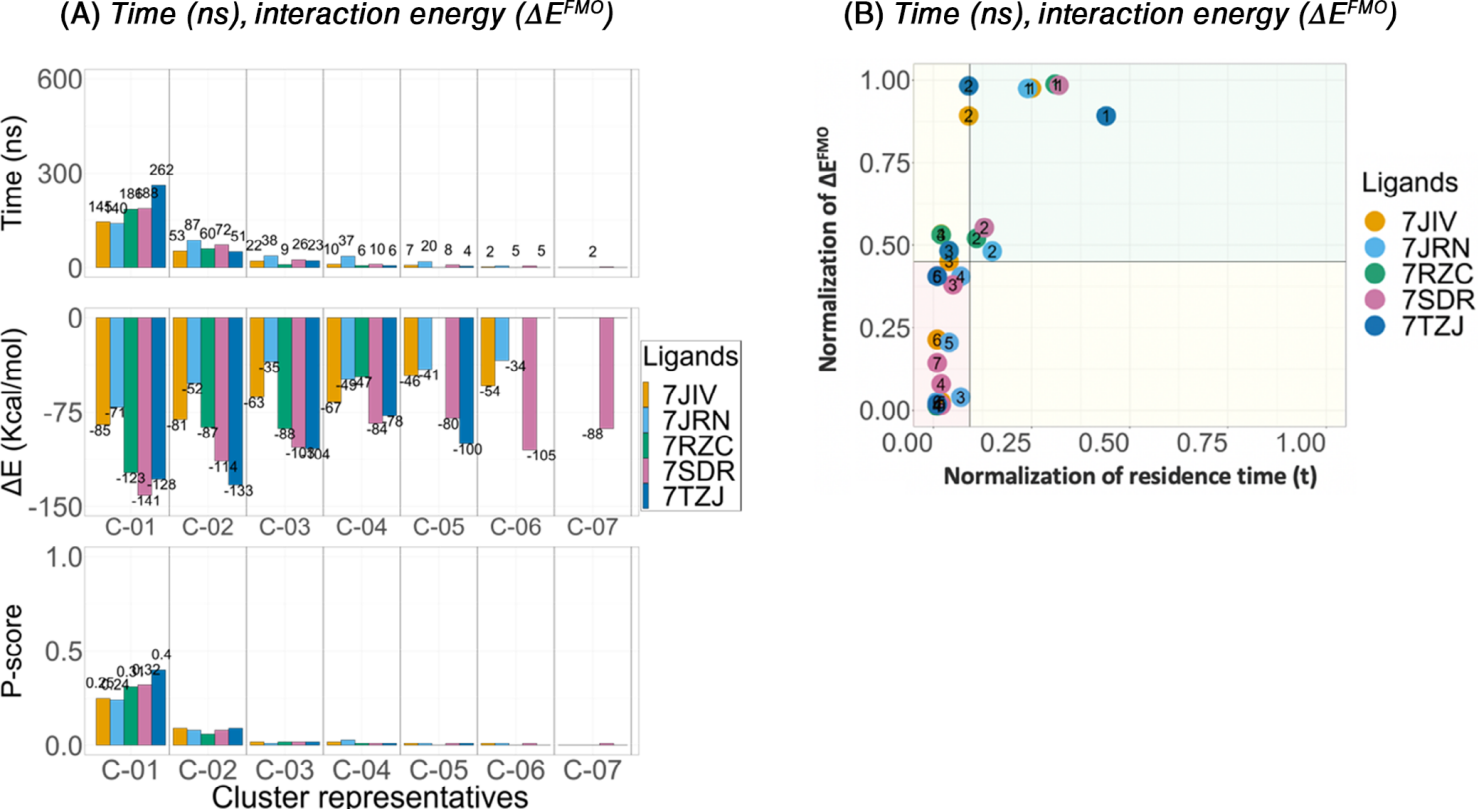
Holo‐closed P‐loop conformation analysis. Clustering of the three replicates for each PL‐pro inhibitor, Lig‐01 (7JIV), Lig‐02 (7JRN), Lig‐03 (7RZC), Lig‐04 (7SDR), and Lig‐05 (7TZJ). For each cluster (C‐0*) the residence time (*t*) (ns), interaction energy (Δ*E* ^FMO^) (kcal/mol) shown on bar plots and *P‐score* diagram.

For the *holo‐closed* P‐loop conformation system, for each cluster of each ligand, the time (ns) the ligand spent stably bound in a certain pose (residence time) and the total average interaction energy (Δ*E* ^FMO^) (kcal/mol) was calculated (Figure 4A).

For the five ligands; Lig‐01 (7JIV) (orange), Lig‐02 (7JRN) (cyan), Lig‐03 (7RZC) (green), Lig‐04 (7SDR) (magenta) and Lig‐05 (7TZJ) (dark blue), cluster representative C‐01 showed the most stable conformation in the binding site, translated by having the longest residence time (t), of 145, 140, 186, 188, and 262 ns, respectively out of the 600 ns concatenated trajectories, rendering the most populated binding pose during the SuMD simulations.

Lig‐03 (7RZC) (green), Lig‐04 (7SDR) (magenta) and Lig‐05 (7TZJ) (dark blue) show a lower IC_50_ values in comparison to Lig‐01 (7JIV) (orange) and Lig‐02 (7JRN) (cyan). In addition, ligands Lig‐03/04/05 spends a longer residence time in the most stable conformation than Lig‐01/02.

The cluster representative C‐01 showed the lowest total average interaction energy (Δ*E* ^FMO^), of −85 (kcal/mol), −71 (kcal/mol), −123 (kcal/mol), −141 (kcal/mol), respectively, for the four ligands: Lig‐01 (7JIV) (orange), Lig‐02 (7JRN) (cyan), Lig‐03 (7RZC) (green), and Lig‐04 (7SDR) (magenta). Thus, for these four ligands, the most stable conformation was calculated to also display the lowest energy, as would be expected. However, for Lig‐05 (7TZJ) (dark blue), although C‐01 appears to be the most stable conformation (the longest residence time (*t*) of 262 ns), it was ranked only as the second lowest total average interaction energy (Δ*E* ^FMO^)‐127.5 kcal/mol after C‐02 with a total average interaction energy (Δ*E* ^FMO^) of −133 kcal/mol.

The *P‐score* was calculated for each cluster of each ligand (Figure 4A,B). The *P‐score diagram* shows that for the five PL‐pro inhibitors Lig‐01 (7JIV), Lig‐02 (7JRN), Lig‐03 (7RZC), Lig‐04 (7SDR), and Lig‐05 (7TZJ), C‐01 falls in the top right corner (green box) formed by the mean of normalization of interaction energy (Δ*E* ^FMO^) and time (t) for all the simulations. The *P‐score* bar‐chart shows that C‐01 for Lig‐01 (7JIV), Lig‐02 (7JRN), Lig‐03 (7RZC), Lig‐04 (7SDR), and Lig‐05 (7TZJ), has the highest values among other poses for each ligand separately (Appendix S1). This indicates that C‐01 is predicted to be the highest‐ranking pose for each ligand.

For the first four ligands, Lig‐01 (7JIV), Lig‐02 (7JRN), Lig‐03 (7RZC), and Lig‐04 (7SDR), C‐01 showed the most stable conformation with the longest residence time (ns) and the lowest total average interaction energy (Δ*E* ^FMO^) (Figure 4A). However, looking for Lig‐05 (7TZJ) (dark blue), although C‐02 showed the lowest binding interaction energy (Δ*E* ^FMO^), yet it shows a low residence time (*t*) in comparison to C‐01, with 51 ns compared to 262 ns for C‐01. The *P‐score* ranks the C‐01 over C‐02 for the lig‐05 (7TZJ) (dark blue).

### 5.3 PL‐pro system: *Apo‐opened* P‐loop

**FIGURE 5:**
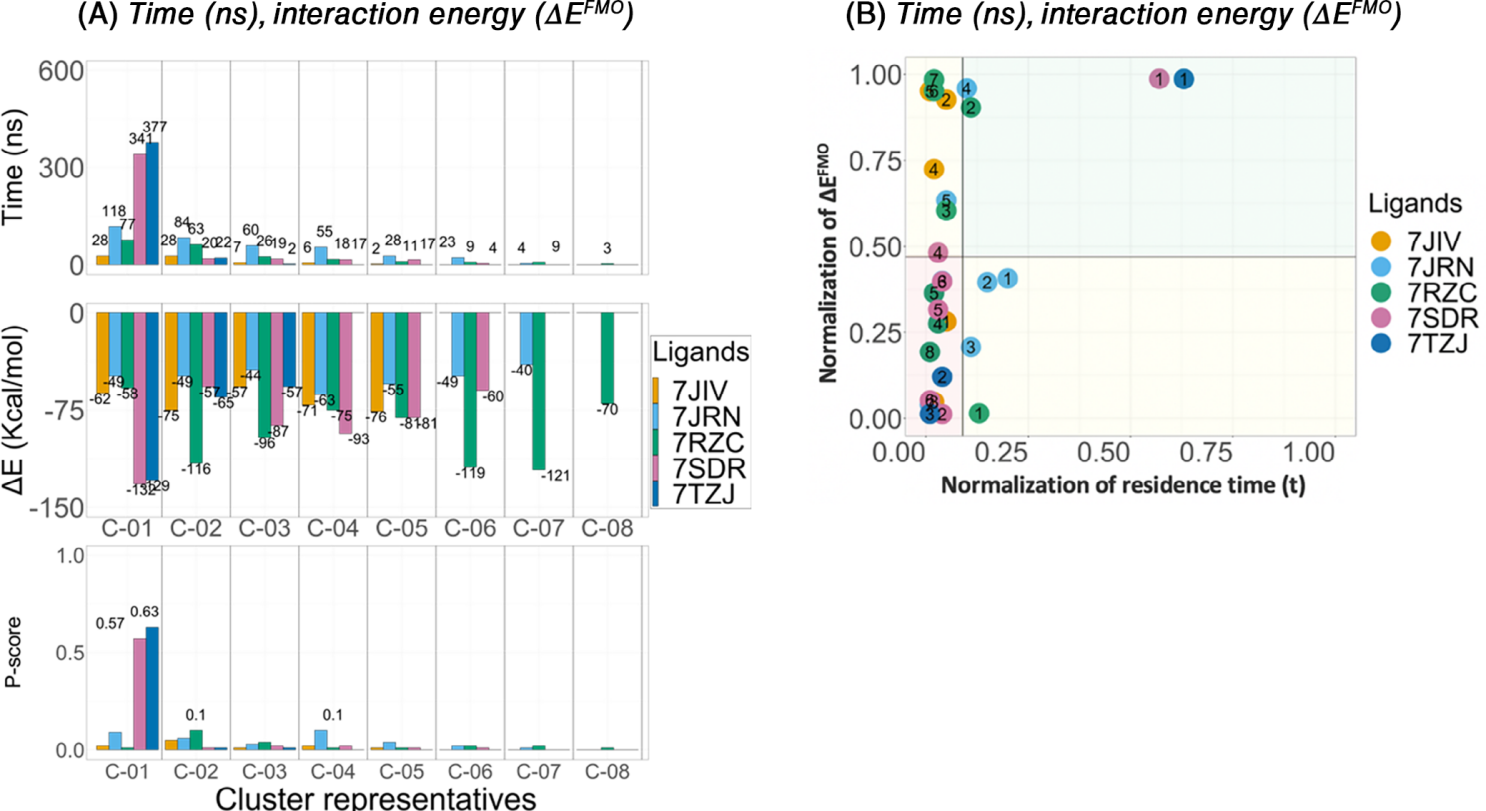
Apo‐opened P‐loop conformation analysis. Clustering of the three replicates for each PL‐pro inhibitor, Lig‐01 (7JIV), Lig‐02 (7JRN), Lig‐03 (7RZC), Lig‐04 (7SDR), and Lig‐05 (7TZJ). For each cluster (C‐0*) the residence time (*t*) (ns), interaction energy (Δ*E* ^FMO^) (kcal/mol) shown on bar plots and *P‐score* diagram.

The same procedures of calculating the time (ns), and interaction energy (Δ*E* ^FMO^) (kcal/mol), for clusters of each ligand, were performed on the *apo‐opened* P‐loop conformation system, (Figure 5A).

For Lig‐01 (7JIV) (orange), the maximum residence times for cluster representatives show a very short residence time of 28 ns found for both C‐01 and C‐02 from a total simulation time of 600 ns. This could be due to the relatively low affinity of 6.4 ± 0.6 μM (Table 1) or indicate that the simulation was not long enough to achieve a stable pose, when starting from the *apo‐opened* P‐loop. When considering the total interaction energy for the ligand, C‐05 showed the lowest interaction energy of −76 kcal/mol.

For Lig‐02 (7JRN) (cyan) although C‐01 showed the longest residence time in the binding site with 118 ns, C‐04 showed the lowest interaction energy with −63 kcal/mol and ranked 4th according to the residence time with 55 ns. For Lig‐03 (7RZC) (green), C‐01 showed the longest residence in the binding site with 77 ns; however, cluster C‐07 showed the lowest interaction energy with a value of −121 kcal/mol. Cluster representative C‐01 for ligands, Lig‐04 (7SDR) (magenta) and Lig‐05 (7TZJ) (dark blue), showed the most stable conformation in the binding site by having the longest residence time (*t*) of 377s and 341 ns respectively. In addition, C‐01 for Lig‐04 (7SDR) and Lig‐05 (7TZJ), showed the lowest total average interaction energy (Δ*E* ^FMO^) of −132 and −129 kcal/mol, respectively.

By calculating the *P‐score* diagram and bar‐chart, indicates that for ligands, Lig‐04 (7SDR) and Lig‐05 (7TZJ), C‐01 is predicted to be the highest‐ranking binding pose of interest with a *P‐score* values of 0.57 and 0.63 respectively, (Figure 5B).

For ligand Lig‐03 (7RZC), the *P‐score* ranks C‐02 as the highest‐ranking pose with a value of 0.1. Although C‐02 did not show the longest residence time in the binding site, with only 63 ns when compared to C‐01 of 77 ns, nor the lowest interaction energy −115 kcal/mol compared to C‐07 with −121 kcal/mol, yet the *P‐score* predicts that the highest‐ranking pose is C‐02.

For Lig‐02 (7JRN), although C‐01 shows the longest residence time (*t*) of 118 ns out of the 600 ns trajectories) (Figure 5A), *P‐score* prioritizes C‐04 as the pose of interest which shows much lower residence time (t) of 55 ns. Also, *P‐score* can identify that C‐02 for Lig‐03 (7RZC) and C‐04 for Lig‐02 (7JRN) yet it also says that both predicted poses are on the border line of residence time (normalization of residence time (Figure 5B), which means, these poses can be significant but prolongation of SuMD simulations is recommended to increase the percentage of confidence in selected poses.

However, for Lig‐01 (7JIV) (orange), the *P‐score* suggests that none of the poses are likely to be prioritized. In the *P‐score* diagram, no points for the Lig‐01 (7JIV) (orange) were found in the top right corner (green box).

### 5.4 PL‐pro system: CMD*‐opened* P‐loop

**FIGURE 6:**
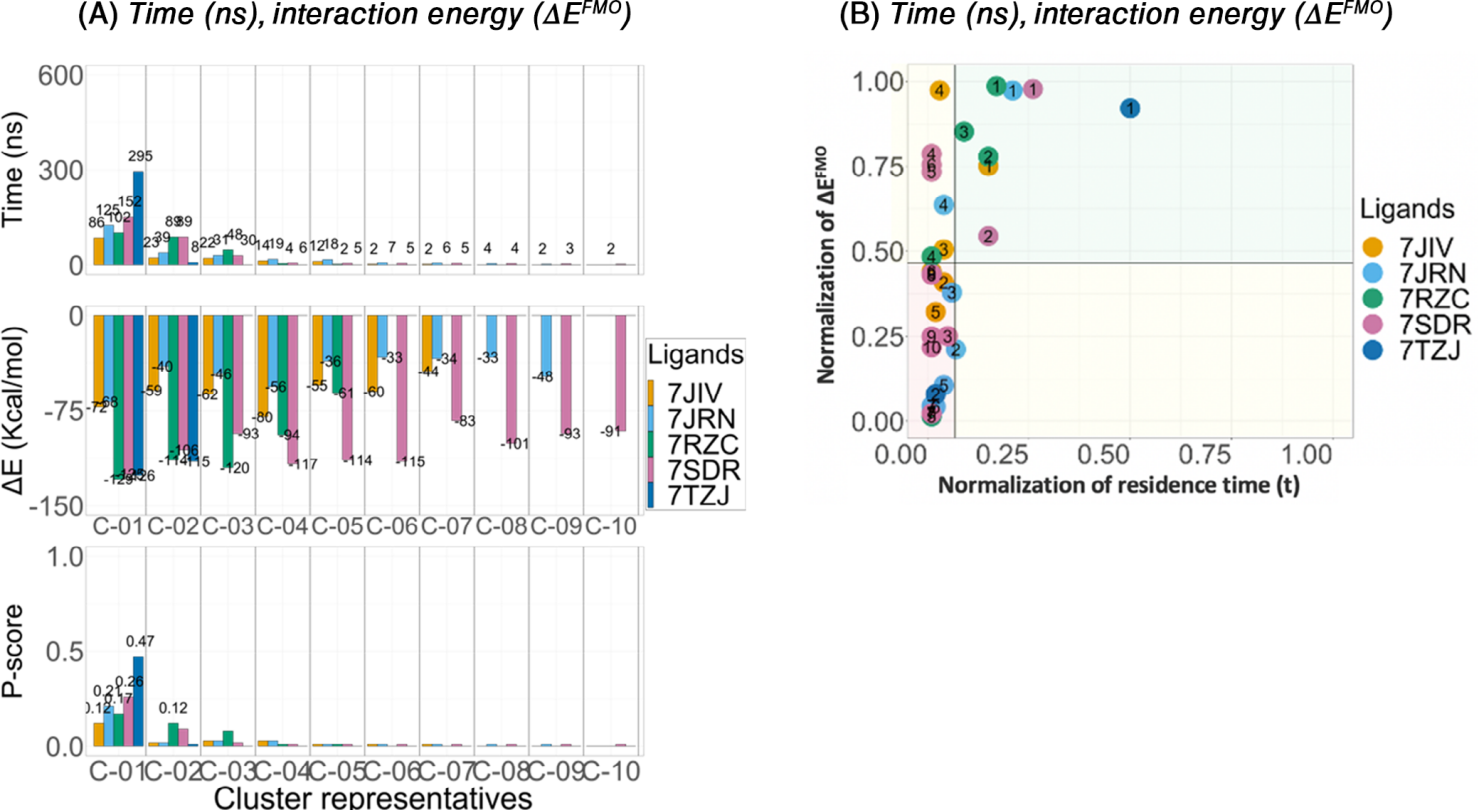
CMD‐opened P‐loop conformation analysis. Clustering of the three replicates for each PL‐pro inhibitor, Lig‐01 (7JIV), Lig‐02 (7JRN), Lig‐03 (7RZC), Lig‐04 (7SDR), and Lig‐05 (7TZJ). For each cluster (C‐0*) the residence time (*t*) (ns), interaction energy (Δ*E* ^FMO^) (kcal/mol) shown on bar plots and *P‐score* diagram.

Starting from the CMD*‐opened* P‐loop conformation system, (Figure 6). For Lig‐01 (7JIV) (orange), C‐01 showed the longest residence time of 86 ns found. This could also be due to the relatively low affinity of 6.4 ± 0.6 μM (Table 1). When considering the total interaction energy for the ligand, C‐04 showed the lowest interaction energy of −80 (kcal/mol). For Lig‐02 (7JRN) (cyan) although C‐01 showed the longest residence time in the binding site with 125 ns, with the lowest interaction energy with −68 kcal/mol. For Lig‐03 (7RZC) (green), Lig‐04 (7SDR) (magenta) and Lig‐05 (7TZJ) (dark blue), C‐01 showed the longest residence in the binding site with 102s, 152, and 295 ns respectively. Also, C‐01 for Lig‐03 (7RZC) (green), Lig‐04 (7SDR) and Lig‐05 (7TZJ), showed the lowest total average interaction energy (Δ*E* ^FMO^) of −129, −125 kcal/mol and −126 kcal/mol, respectively.

According to the *P‐score* diagram and bar‐chart, indicates that for all ligands, C‐01 is predicted to be the highest‐ranking binding pose of interest with a *P‐score* values of 0.12, 0.21, 0.17, 0.26, and 0.47 respectively. Although C‐01 did not show the lowest total average interaction energy, for ligands Lig‐01 (7JIV) (orange), and Lig‐02 (7JRN) (cyan).

## 6 VALIDATION OF PREDICTED POSES BY *P‐score*

**FIGURE 7:**
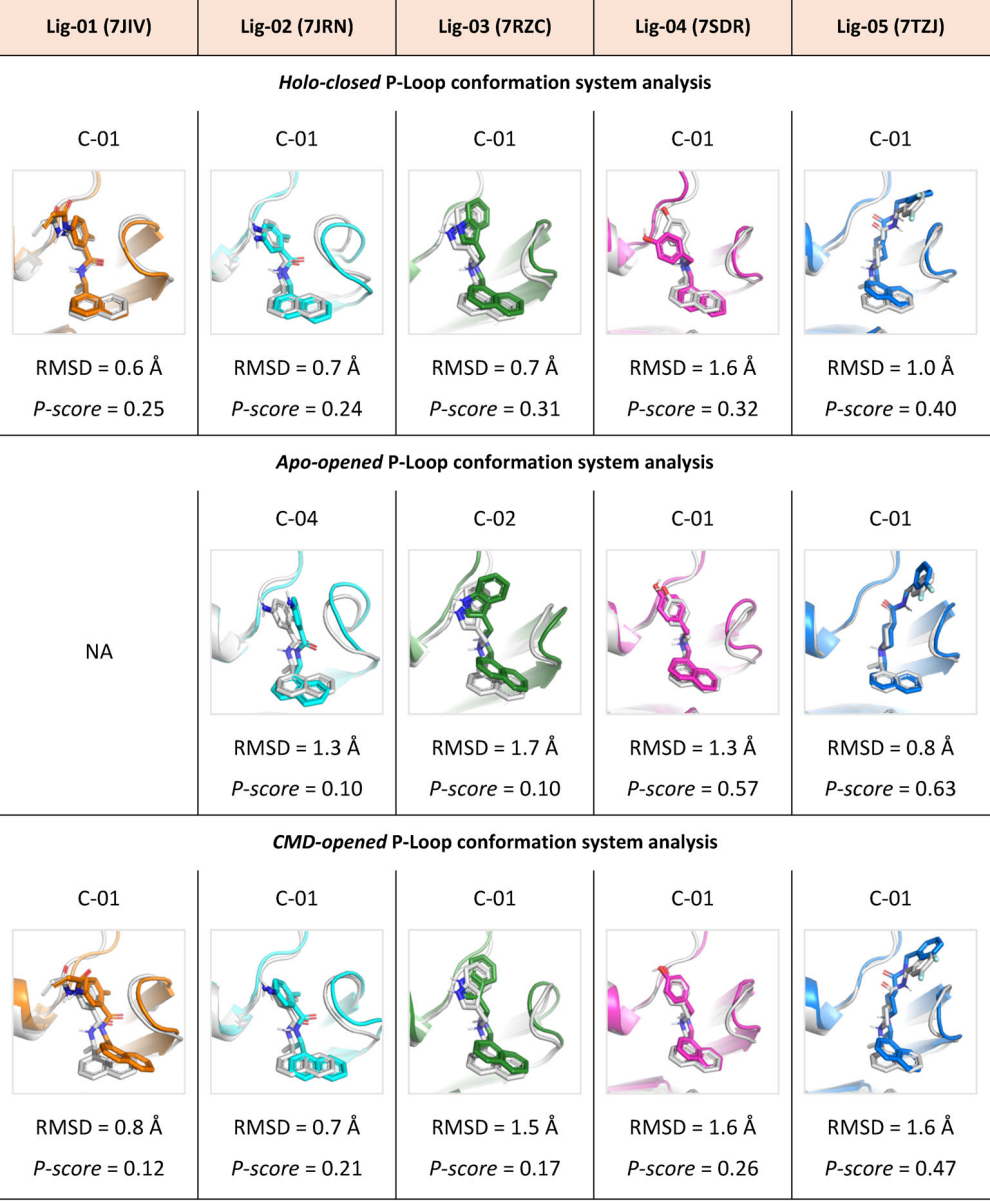
Root‐mean‐square deviations of clusters with the highest *P‐score* value for each PL‐pro inhibitor starting from the three different binding sites conformations, Lig‐01 (7JIV), Lig‐02 (7JRN), Lig‐03 (7RZC), Lig‐04 (7SDR), and Lig‐05 (7TZJ), against its correspondent reference crystal structure (white). NA, not available, since *P‐score* did not prioritize any pose during the simulation.

The accuracy of the *P‐score* predictions was then assessed by comparing the highest *P‐score* structure for each ligand with its respective crystallographic reference, computing the RMSD of non‐hydrogen atomic coordinates. Selected binding poses for each ligand in each SuMD simulation system, starting from both *holo‐closed* or *apo‐opened* P‐loop conformation, shows an RMSD from the reference structure that falls below 2 Å, with the lowest being 0.6 Å and the highest of 1.6 Å, (Figure 7).

This indicates a good prediction of the correct binding pose for each ligand. The selected pose for each ligand also established nearly the same interaction energy (Δ*E* ^FMO^) against the binding site residues of its corresponding crystalized pose (reference pose). To have a further investigation on the predicted binding poses, detailed protein–ligand binding analysis is performed on the interaction with the binding site residues (Appendix S1 (3–8)).

## 7 PROTOCOL BENCHMARKING

Simple docking and induced‐fit docking were evaluated as a basis of comparison for the binding pose prediction of the five ligands in terms of different scoring functions, against the three P‐loop conformation, the *holo‐closed*, *apo‐opened* and *CMD‐opened* conformations.

**FIGURE 8:**
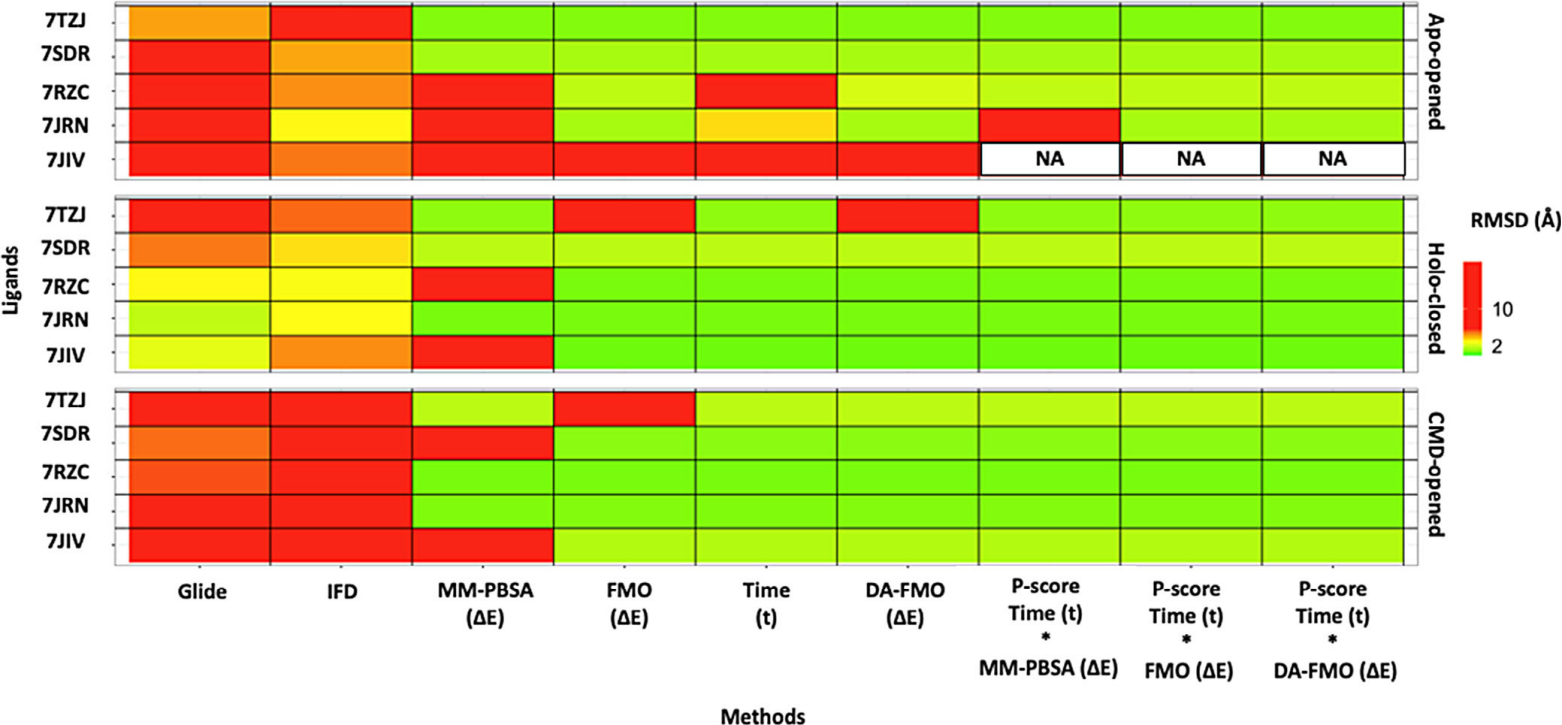
Heatmap of root‐mean‐square deviation (RMSD) with a threshold of 2 Å, showing lowest RMSD reach by each ligand, Lig‐01 (7JIV), Lig‐02 (7JRN), Lig‐03(7RZC), Lig‐04 (7SDR), and Lig‐05 (7TZJ) (*y*‐axis), against different method used, Glide (simple docking using glide and docking scoring function), IFD (induced fit docking, scoring using docking scoring function), MM‐PBSA (Δ*E*) (MM‐PBSA (Δ*E*) binding energy used for prediction), time (*t*) (using time [*t*] for residence), DA‐FMO (Δ*E*) (using interaction energy of DA‐QM‐FMO as a single descriptor for affinity). NA, not applicable.

Simple docking using Glide and its docking score function was successful in generating the correct binding pose (RMSD <2 Å) of only Lig‐01 (7JIV) and Lig‐02 (7JRN) in the *holo‐closed* P‐loop conformations. However, simple docking as well as induced‐fit docking failed to predict the correct binding pose for the five ligands in both *apo‐opened* and *CMD‐opened* P‐loop conformations (RMSD >2 Å) (Figure 8).

However, by utilizing the SuMD approach, we were able to generate the correct binding pose independent from the binding site conformation except for the ligand Lig‐01 (7JIV) in the *apo‐opened* simulation. These latter results may be due to the ligand's low affinity with the open conformation of the binding site, probably indicating the need for longer simulation times. By contrast, when MM‐PBSA (Δ*E*) was applied on the clusters from the SuMD, it showed low accuracy in predicting the correct binding pose with 50% correct predictions.

Taking the residence time (*t*) or the interaction energy DA‐FMO (Δ*E*) alone as a single descriptor to account for the differentiation between binding poses, the most stable conformation as predicted from the SuMD was also the lowest energy conformation as predicted by DA‐QM‐FMO or FMO (on a single snapshot) in most cases.

However, in some cases, either one or both metrics did not predict the experimental binding pose. This is likely due to the limited simulation time during SuMD, and the fact that FMO accounts for the interaction energy between ligand and binding site residues but does not calculate the binding free energy of the system on atomic level. Also, FMO calculations rely on a basis set of atomic orbitals to describe the electronic structure of the system. However, if the basis set is not sufficiently large, important electronic interactions between fragments may be missed, leading to errors in the predicted properties.

Applying the *P‐score* matrix, using the residence time and the binding energy from MM‐PBSA, FMO or DA‐FMO, was apply to accurately rank order generated poses for all ligands independent from the starting point of the binding site. Also, in the case of 7JIV in the apo‐opened conformation simulation, the *P‐score* was able to identify it as “NA” (Not available) which refers to no poses were prioritized in these simulations, eliminating the false positive predictions. A single false prediction by the *P‐score* is found with the ligand 7JRN in the Apo‐opened, however this is because MM‐PBSA as energy calculation is based on molecular mechanics which is less accurate than quantum‐level FMO, therefore using less accurate methods to provide descriptors for the *P‐score* matrix can results in false positives.

### 7.1 M‐pro target system

A second SARS‐CoV‐2 protease protein is Mpro which is one of two cysteine viral proteases essential for viral replication. We apply the same procedures mentioned for PL‐pro on six different ligands co‐crystalized with the Mpro protein with PDB IDs: Lig‐01 (5R81), Lig‐02 (5R83), Lig‐03 (5R84), Lig‐04 (5RE4), Lig‐05 (5RF1), and Lig‐06 (5RF3).^58^ In this case, each ligand was subjected to different sampling time using SuMD, with a total simulation time of 257 ns all‐combined.

**FIGURE 9:**
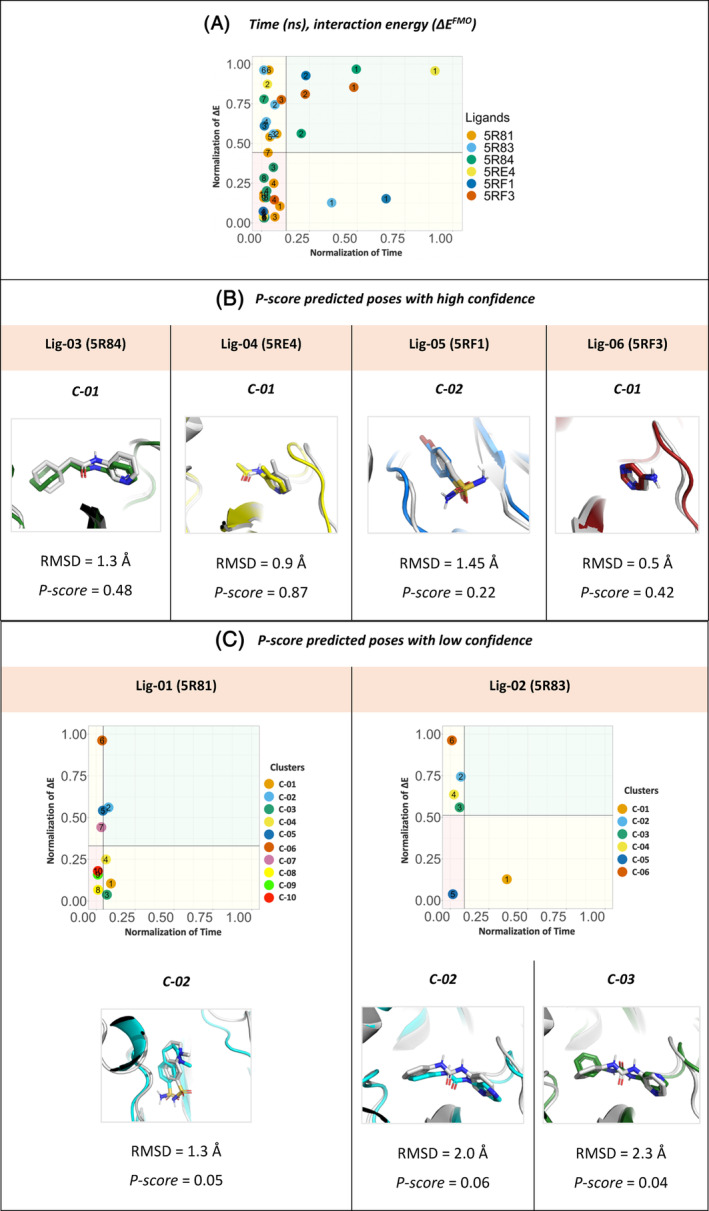
(A) *P‐score* diagram for Mpro target screening. Clusters representative for PDB IDs: 5R81, 5R83, 5R84, 5RE4, 5RF1, and 5RF3. For each cluster numbered, and the residence time (*t*) (ns), interaction energy (Δ*E* ^FMO^) (kcal/mol) shown on bar *P‐score* diagram *x* and *y*‐axis respectively. (B) Root‐mean‐square deviation (RMSD) of clusters with the highest *P‐score* value for each M‐pro binders, Lig‐03 (5R84), Lig‐04 (5RE4), Lig‐05 (5RF1), and Lig‐06 (5RF3), against its correspondent reference crystal structure (white). (C) *P‐score* diagram for Mpro target screening. RMSD of clusters with the low *P‐score* confidence value for each M‐pro binders, Lig‐01 (5R81), Lig‐02 (5R83), against its correspondent reference crystal structure (white).

*P‐score* quadrant matrix was preformed using the residence time and QM‐FMO, to predict the binding pose for each ligand, (Figure 9A). In relative comparison between all simulations combined, *P‐score* prioritized a binding pose for ligands; Lig‐03 (5R84), Lig‐04 (5RE4), and Lig‐06 (5RF3), represented in clusters numbers C‐01, and for Lig‐05 (5RF1) cluster number C‐02, were identified as the poses of interest. Poses were accurately predicted in the four prioritized ligands shows a value of (RMSD <2 Å) against the reference crystal structure. However, for the ligands Lig‐01 (5R81), and Lig‐02 (5R83), *P‐score* did not prioritize any of the poses that have been identified in relative comparison to the rest of the ligands. By treating each of the screened molecules separately, (Figure 9C; Appendix S1), *P‐score* identify for ligands, Lig‐01 (5R81), cluster number C‐02 as potential pose of interest, while for Lig‐02 (5R83), both C‐02 and C‐03 falls on the mean line that intersect the residence time.

Accordingly, *P‐score* in these three cases is prioritizing the mentioned poses per ligand but with lower confidence than the first four ligands. Therefore, *P‐score* is suggesting that these are the potential poses of interest, but more simulation time is needed to assure the prediction. However, assessing the RMSD in respect to the co‐crystalized reference, we can find that *P‐score* prioritized poses even with low confidence, have a value of (RMSD <2.5 Å), where the ligands bind in a well‐defined orientation in respect to the reference pose, where these poses are found to be not the lowest binding interaction energy nor the longest residence time, yet *P‐score* prioritized the nearest poses to their corresponding reference structure.

### 7.2 *P*. *aeruginosa* LpxC— *P‐score* using MMPBSA

**FIGURE 10:**
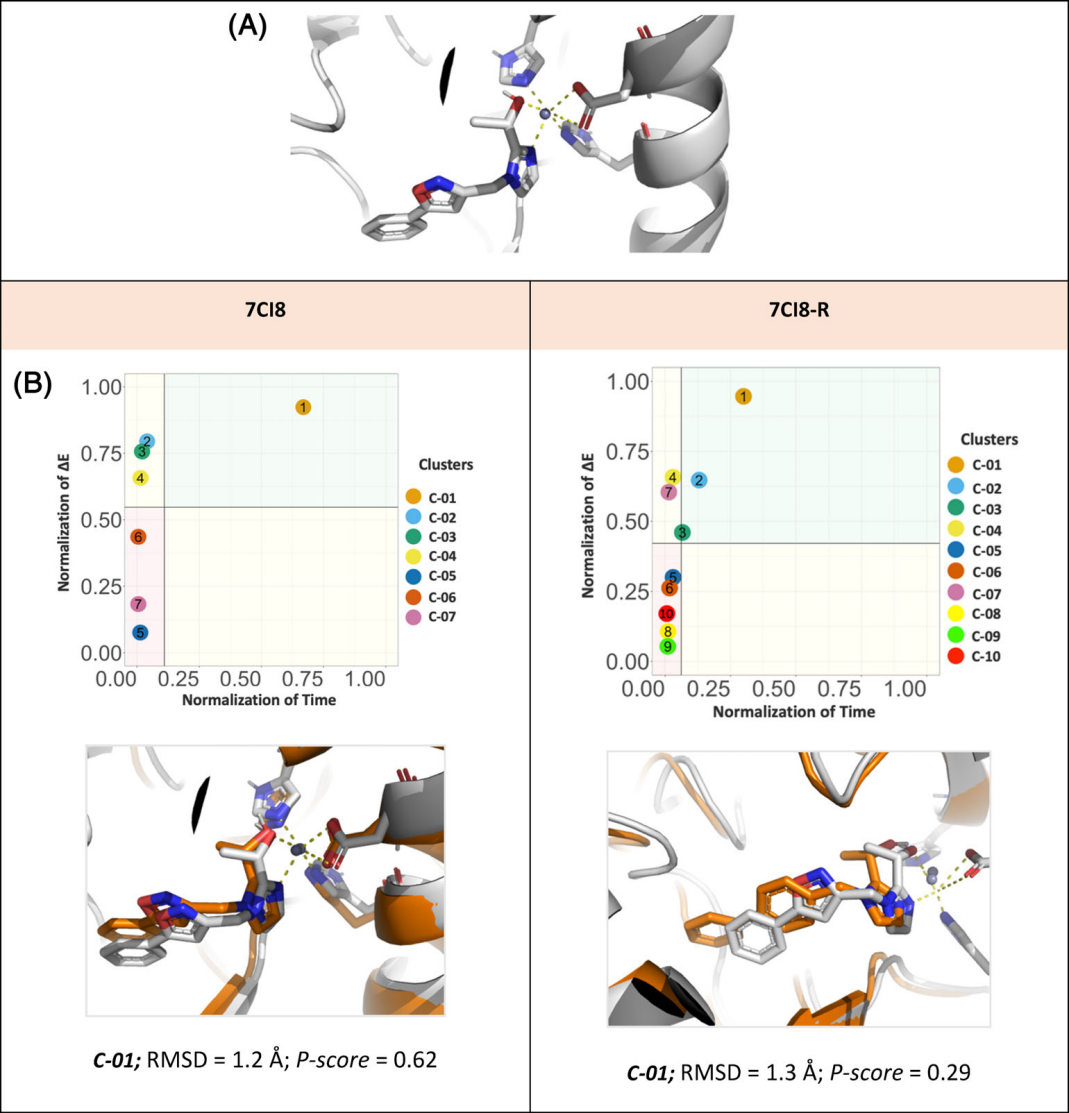
(A) *Pseudomonas*. *aeruginosa* LpxC Zinc binding site, Zn^2+^, between ASP‐241, HIS‐237 and HIS‐78. (B) *P‐score* diagram for LpxC target. RMSD of Clusters with the low *P‐score* confidence value for each M‐pro binders, 7CI8, 7CI8‐R, against its correspondent reference crystal structure (white).

*P*. *aeruginosa* LpxC, is a zinc metalloenzyme that catalyzes the first step in the biosynthesis of Lipid A, an essential component of the cell envelope of Gram‐negative bacteria.^59^ Its binding site is formed of a 4‐coordinated zinc metal centers with 3 residues; ASP‐241, HIS‐237, and HIS‐78, (Figure 10A). In this example, the ligand is sampled within the binding site, however for energy calculations MM‐PBSA was used instead of DA‐QM‐FMO, due to the positive charge of the Zn^2+^‐ion, that can be challenging for QM‐approaches. Therefore, the *P‐score* matrix, is built around residence time (*t*) and the binding energy calculation from MM‐PBSA. This protein is found in a complex structure with PDB ID; 7CI8, where there have been many modifications to the core scaffold and R‐groups of this molecule reported in the literature.^56^ We apply the *P‐score* protocol on the crystalized ligand and one of its core scaffold modifications (referred as 7CI8‐R).^59^

In both ligands, the 7CI8 and 7CI8‐R, the *P‐score* has a high confidence in the binding pose predicted, represented in cluster number C‐01. The 7CI8 ligand has a well aligned conformation with Zn^2+^ ion binding interaction, reflected in the RMSD of 1.2 Å with the reference molecule. On the other hand, for the 7CI8‐R as there is no crystal structure, however they share the same common head that is binding to the Zn^2+^ ion, RMSD was calculated only on the atom pair with the reference molecule, which shows also a well aligned Zn^2+^‐ion binding and an RMSD of 1.3 Å. Figure 10B.

### 7.3 Further study cases; heat shock protein 90, p38 kinase, and myeloid cell leukemia 1

Heat shock protein 90 (Hsp90) plays a key role in stress response and protection of the cell against the effects of mutation, p38 Kinase (p38), and Myeloid Cell Leukemia 1 (Mcl‐1) a member of the Bcl‐2 family of proteins, is overexpressed and amplified in various cancers and promotes the aberrant survival of tumor cells that otherwise would undergo apoptosis. These targets are for treatment of many diseases. For the Hsp90, p38 and Mcl‐1 the PDB IDs; 3FT5. 1W7H and 4HW3 were used as representative targets with their co‐crystalized ligands.^60^, ^61^, ^62^

**FIGURE 11:**
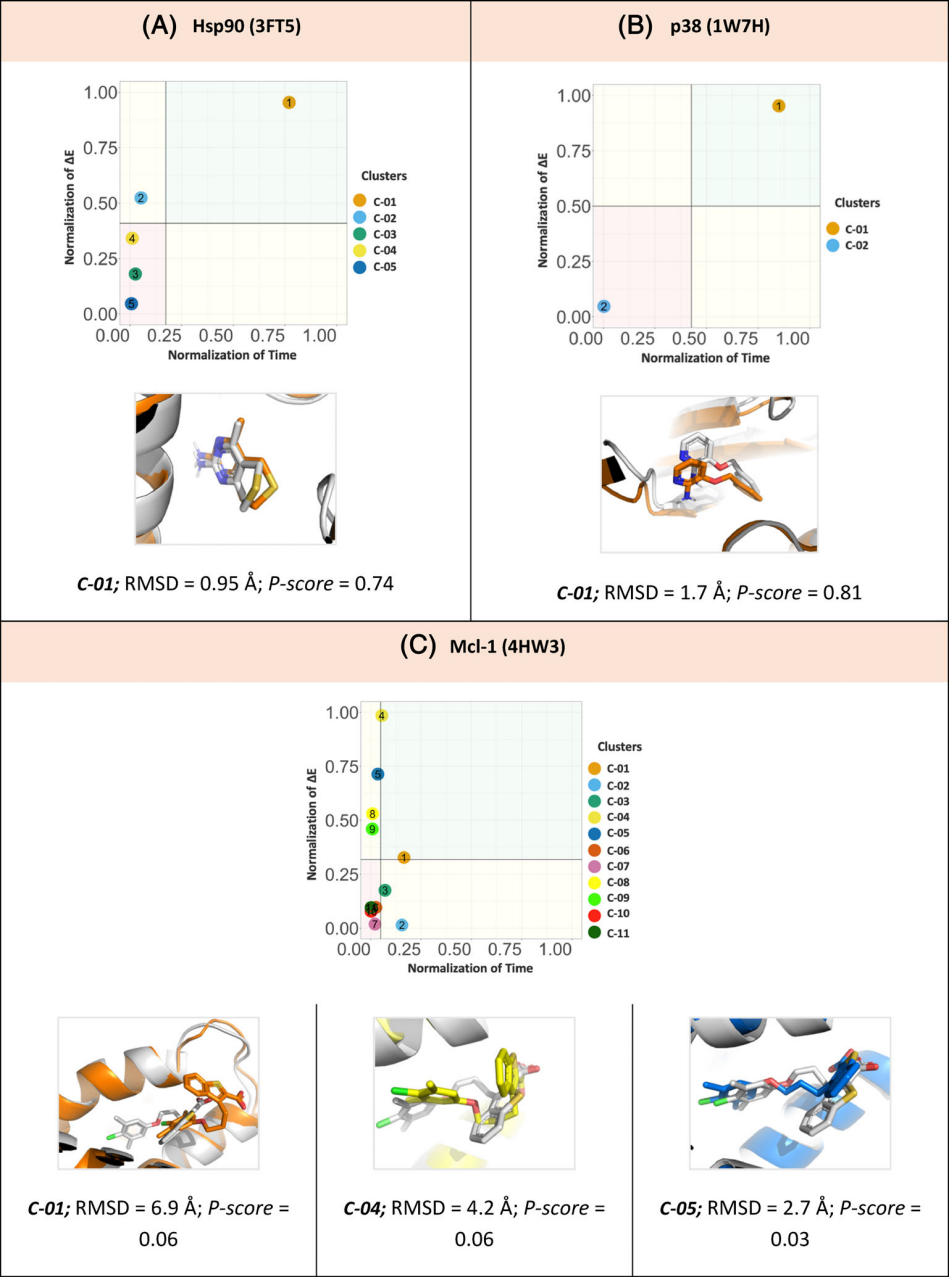
(A,B) *P‐score* diagram for Hsp90 and p38 targets. root‐mean‐square deviation (RMSD) of clusters with the low *P‐score* confidence value for both, Hsp90 (3FT5), p38 (1W7H), against its correspondent reference crystal structure (white). (C) *P‐score* diagram for Mcl‐1 target. RMSD of Clusters with the low *P‐score* confidence value for Mcl‐1 (4HW3), against its correspondent reference crystal structure (white).

As shown in (Figure 11A,B), *P‐score* prioritized a pose for both targets the Hsp90 and p38, where cluster number C‐01 showed high confidence, that is reflected in a high *P‐score* value of 0.74 and 0.81, respectively. Compared to the RMSD value of the co‐crystalized poses, both ligands; Hsp90 and p38 shows a well‐established superposition on the crystalized reference with RMSD 0.95 Å and 1.70 Å, respectively. For the case of Mcl‐1, *P‐score* matrix shows two different clusters C‐02 and C‐03 on the mean value of residence time (t) and C‐01 on the mean value of interaction energy (Figure 11C). Although the pose represented by C‐03 is with acceptable range of RMSD value 2.7 Å, it is recommended to increase the sampling phase to allow more exploration of local minima around the protein–ligand binding.

### 7.4 Correlation between interaction energy (kcal/mol) and residence time ( *t* )

Pearson correlation coefficient (*r*) was calculated for each ligand simulation independently based on the simulation data, (as detailed in the Appendix S1; 9).^63^ Our analysis reveals a consistent trend; when the sampling time is long enough to allow the ligand to adequately explore the binding site and assume stable conformations across various local minima, a negative correlation emerges between prolonged residence time (t) and lower interaction energy (kcal/mol). This correlation suggests that; as the energy of interaction decreases, the residence time of the ligand tends to increase, indicating a more stable binding. This observation is supported by a high confidence level in the prediction, as indicated by the associated p‐values. However, in simulations with insufficient dynamic sampling time, correlation is barely predicted and mostly not found, yet the *P‐score* can accurately prioritize the correct binding pose by combining both descriptors in a normalization equation. These findings underline the significance of both descriptors; residence time and interaction energy, in characterizing protein–ligand binding dynamics, and thus affirming the principles outlined in the *P‐score* theory.

### 7.5 *P‐score* for hit identification

The experimental Free energy (Δ*G*_exp_) for the IC50, as shown in Equation (4):

(4) $$∆G_{\exp}=R\times T\times \mathrm{Ln}\;\left(\mathrm{IC}50\right).$$

Experimental free energy equation, *R* and *T* represent the gas constant and temperature, respectively.

The *P‐score* for the experimental value can be calculated by considering the normalization of the simulation time; *t*_*i*_/*T* = 1, since the crystal structure represents a static reference pose.

### 7.6 PL‐pro system

**FIGURE 12:**
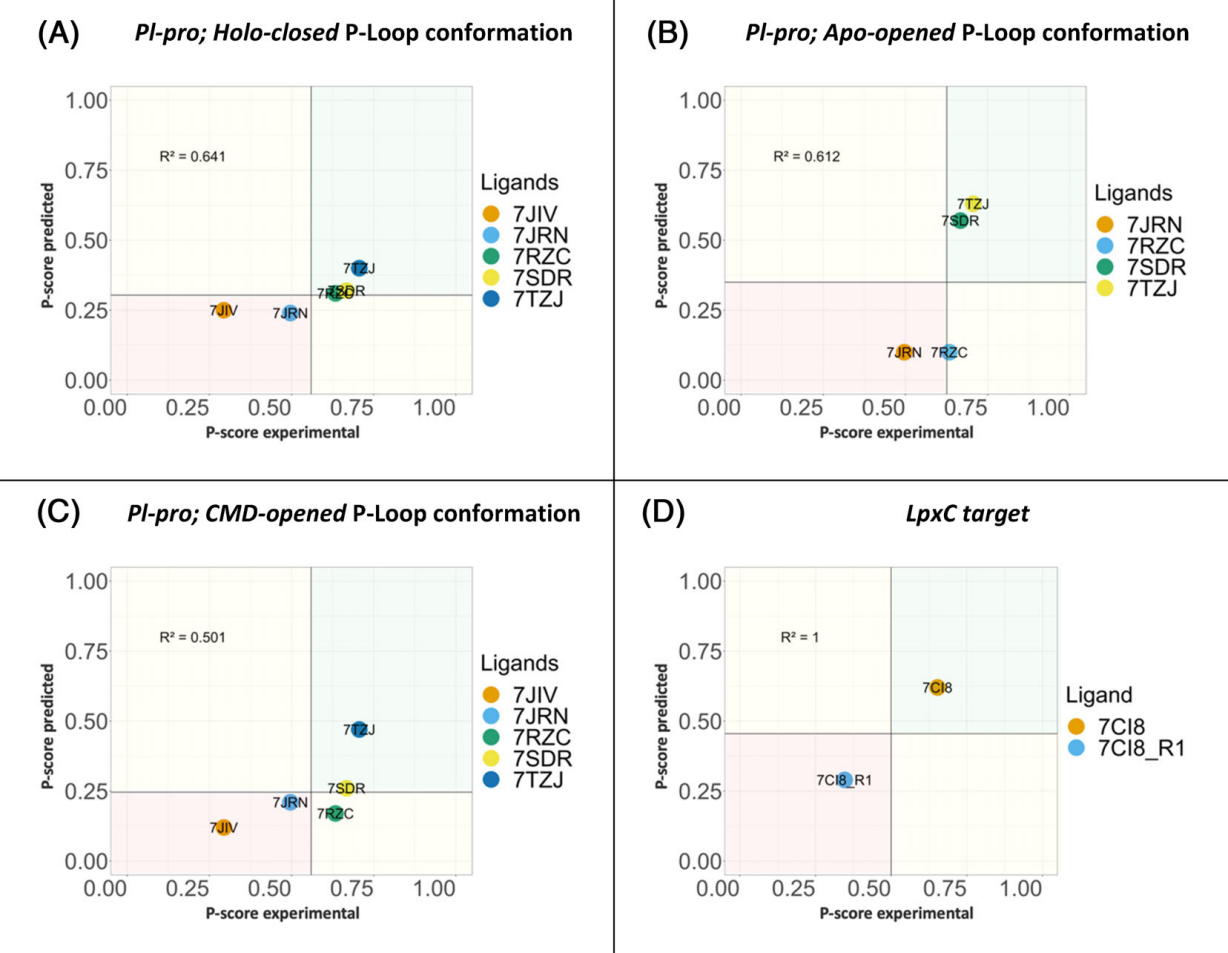
*P‐score* against normalization of delta G experimental for the five ligands of PL‐pro inhibitors, Lig‐01 (7JIV), Lig‐02 (7JRN), Lig‐03 (7RZC), Lig‐04 (7SDR), and Lig‐05 (7TZJ). Starting from, (A) Holo‐closed. (B) Apo‐opened. (C) CMD‐opened P‐loop conformation systems. (D) LpxC target.

Starting from the *holo‐closed* P‐loop conformation system, the *P‐score* predicted against *P‐score* experimental, identify Lig‐03 (7RZC), Lig‐04 (7SDR), and Lig‐05 (7TZJ) as the most active hits when compared to the Lig‐01 (7JIV) and Lig‐02 (7JRN) (Figure 12A).

Starting from the *apo‐opened* P‐loop conformation system the *P‐score* identifies the Lig‐04 (7SDR) and Lig‐05 (7TZJ), as the most active hits. For Lig‐03 (7RZC), as mentioned earlier, prolongation of the simulation is needed to increase the confidence of the binding pose. Lig‐02 (7JRN) falls in the less potent (red box) left bottom corner of the graph, and for Lig‐01 (7JIV) no poses were identified by the *P‐score*, (Figure 12B).

Starting from the CMD‐opened P‐loop conformation *P‐score* ranks Lig‐04 (7SDR), and Lig‐05 (7TZJ) as the most active hits when compared to the Lig‐01 (7JIV) and Lig‐02 (7JRN) and Lig‐03 (7RZC), same as in *apo‐opened* P‐loop conformation system (Figure 12C). These results corelated with the experimental IC_50_ values showing that the difference in potency between molecules Lig‐03 (7RZC), Lig‐04 (7SDR), and Lig‐05 (7TZJ) and molecules Lig‐01 (7JIV), and Lig‐02 (7JRN) can be identified as more potent hits in comparison to molecules Lig‐01 (7JIV), and Lig‐02 (7JRN).

Also, there you can see a correlation between the *P‐score* predicted and the *P‐score* experimental, in the three cases, showing that the holo‐closed conformation has the highest correlation of 0.64 *R* ^2^ compared to the Apo‐opened and CMD‐opened P‐loop systems conformations. This can conclude that the more stable the binding conformation is the better the correlation of the *P‐score* predicted with the *P‐score* experimental, as in a wider open binding site conformation, the system needs more sampling time to stabilize a local‐minima with a binding interaction.

### 7.7 LpxC system

Both molecules in the protocol mentioned above have reported IC50 values (7CI8; IC50 = 0.88 nM and 7CI8‐R; IC50 = 3.94 mM). Although MM‐PBSA energy calculation showed that the 7CI8‐R binding energy is −23.9 (kcal/mol), and 7CI8 binding energy is −21.7 (kcal/mol), which is not reflecting the correct correlation with the experimental IC50s. However, combining the residence time (t) using the *P‐score* predicted shows a very good correlation to distinguish between the μM and nM range molecules, showing that 7CI8 falls in the green box while the μM range 7CI8‐R is in the red box. (Figure 12D).

## 8 CONCLUSION

The Identification of a suitably representative binding pose for a ligand in a protein pocket is challenging as it is a complex process. Here we introduce an approach using supervised molecular dynamics, coupled with scoring the binding poses using energy calculation methods; Dynamical Averaging of Quantum Mechanics Fragment Molecular Orbital (DA‐QM‐FMO), FMO or MM‐PBSA.

Our studies highlight the approach could be further enhanced by combining the trajectories of the supervised molecular dynamics with the calculated binding energy using DA‐QM‐FMO as the *P‐score*. When using both the time of the ligand in any binding pose and the calculated binding energy are considered as descriptors and normalized, giving a score between 0 and 1. The *P‐score* was able to predict correctly the experimental binding poses with good accuracy, independent from the ligands starting point or the binding site conformation. In addition, it was able to predict when none of the correct binding poses were found to be the experimental binding pose. Although each of the mentioned case studies have their complexity, PL‐pro was a particularly challenging target, due to the flexibility of the active site and the very flexible P‐loop which closes over the ligand.

Although there is an increase in time and cost using SuMD compared to Glide and docking strategies. Considering the flexible nature of protein targets is crucial for predicting the binding events hence the introduction of dynamics is a necessity, even though the cost can be a limitation that needs careful consideration for future perspectives.

## Supporting information


**Appendix S1:** Supplementary Information.

## Data Availability

The data that supports the findings of this study are available in the supplementary material of this article.
