# Supplementary material for: Accurate prediction of dynamic protein–ligand binding using P‐score ranking
Source: J Comput Chem. 2024 Apr 22;45(20):1762–78. doi: 10.1002/jcc.27370 (PMC11980828; doi:10.1002/jcc.27370)
Supplement: Supplementary file 1 — Appendix S1: Supplementary Information. [file JCC-45-1762-s001.zip › supplementry_files.pdf]

Supplementary data

A. ***Holo-closed*** **P-Loop conformation system**

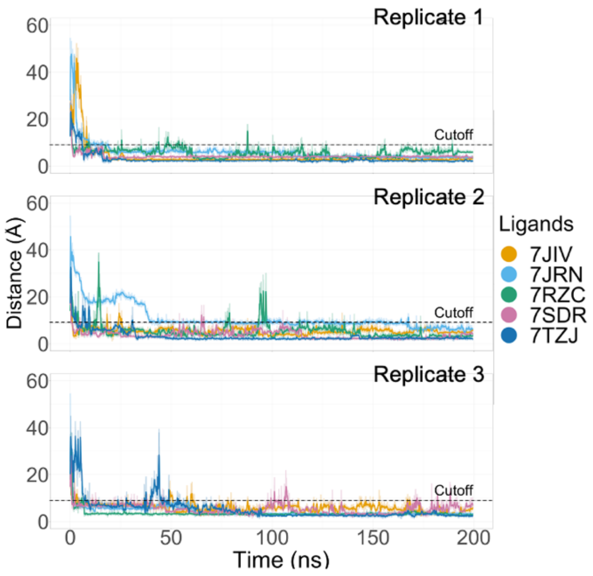

B. ***Apo-opened*** **P-Loop conformation system**

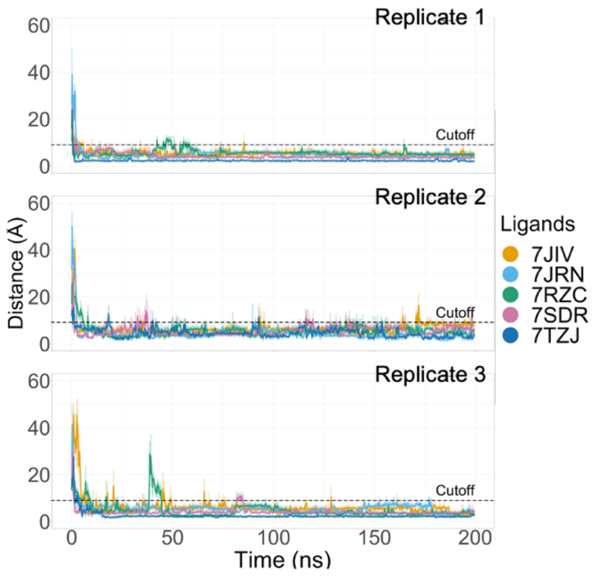

C. ***CMD-opened*** **P-Loop conformation system**

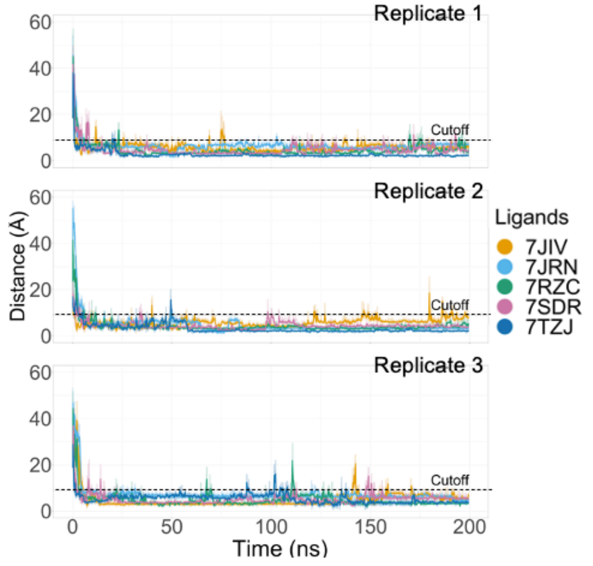

Supplementary (1): *The 3 replicates of SuMD simulation for each PL-pro inhibitor, Lig-01 (7JIV), Lig-02 (7JRN), Lig-03 (7RZC), Lig-04 (7SDR), and Lig05 (7TZJ). Starting from, the* **A.** *Holo-closed P-loop conformation system*, **B.** *Apo-opened P-loop conformation system.* **C.** *CMD-opened P-loop conformation system. (Cut-off = 9 Å)*

P-score values															
System	***Holo-closed* P-Loop conformation**							***Apo-opened* P-Loop conformation**							
clusters	C1	C2	C3	C4	C5	C6	C7	C1	C2	C3	C4	C5	C6	C7	C8
7JIV Lig-01	0.25	0.08	0.02	0.01	0.01	0.01		0.02	0.04	0.01	0.02	0.01			
7JRN Lig-02	0.24	0.08	0.01	0.03	0.01	0.01		0.09	0.06	0.03	0.10	0.04	0.02	0.01	
7RZC Lig-03	0.31	0.06	0.02	0.01				0.01	0.10	0.04	0.01	0.01	0.02	0.02	0.01
7SDR Lig-04	0.32	0.08	0.02	0.01	0.01	0.01	0.01	0.57	0.01	0.02	0.02	0.01	0.01	0.01	0.01
7TZJ Lig-05	0.40	0.09	0.02	0.01	0.01			0.63	0.01	0.01					

***Supplementary (2), Table 2:* P-score values for each cluster representative for both systems; Holo-closed and Apo-opened P-Loop conformations**

Holo-closed* P-Loop conformation system - *Lig-01 (7JIV)

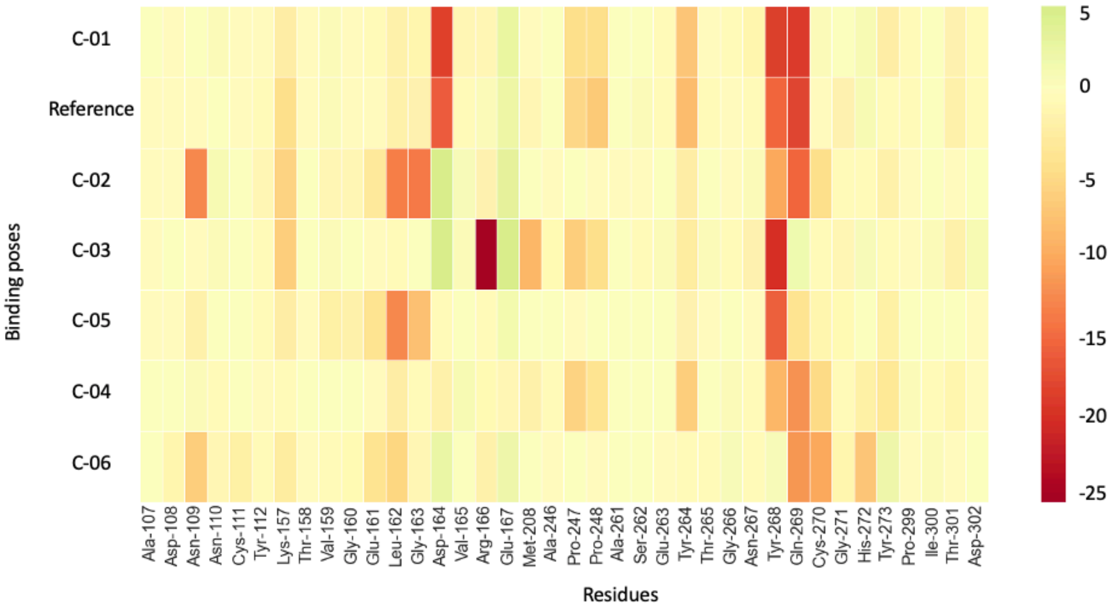

C0-1	C0-2	C0-3	C0-4	C0-5	C0-6
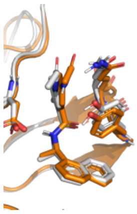	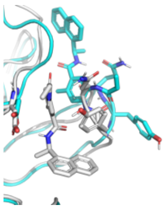	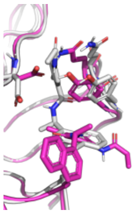	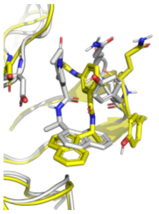	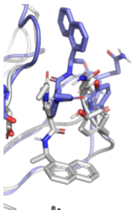	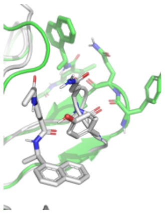
RMSD = 0.60 Å	RMSD = 9.40 Å	RMSD = 8.20 Å	RMSD = 5.30 Å	RMSD = 7.80 Å	RMSD = 12.9 Å
P-score = 0.25	*P-score* = 0.09	*P-score* = 0.02	*P-score* = 0.02	*P-score* = 0.01	*P-score* = 0.01

Supplementary (3a)*, *Lig-01 (7JIV) *interaction energy (ΔE^{FMO}) (Kcal/mol) were calculated and shown on heatmap from Holo-closed SuMD simulations, against the binding site residues, for ligand Lig-01 PDB ID; 7JIV. RMSD of each pose was calculated against the heavy atoms of reference (crystal structure). P-score represents the prioritized pose.*

Apo-opened P-Loop conformation system - _Lig-01 (7JIV)_

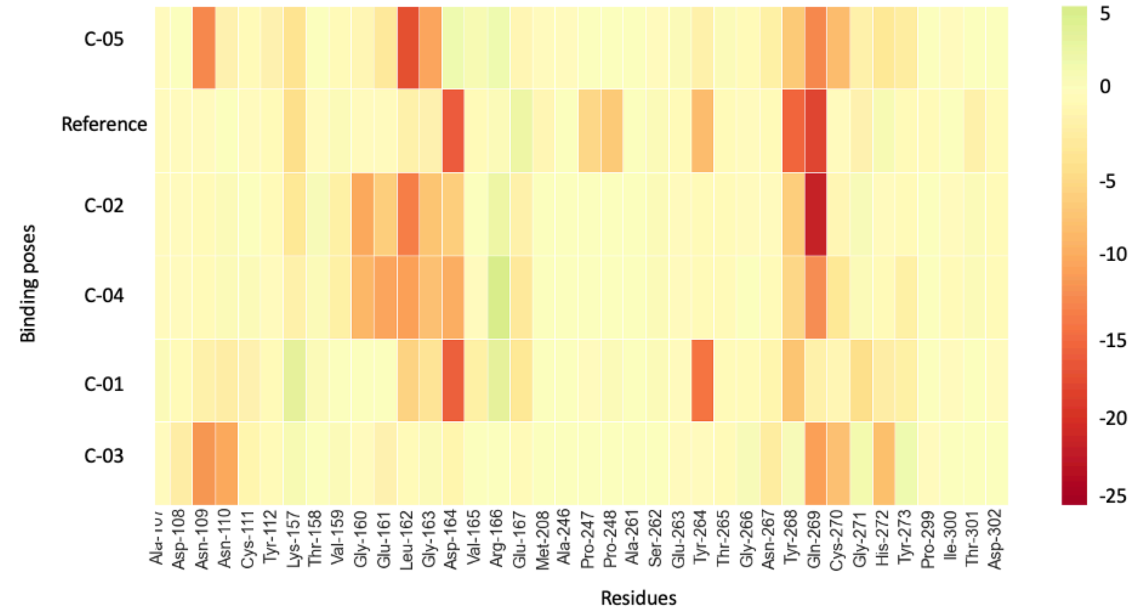

C0-1	C0-2	C0-3	C0-4	C0-5
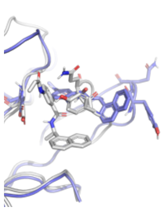	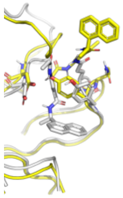	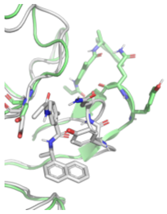	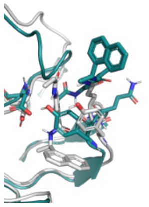	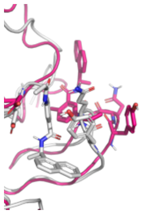
RMSD = 8.15 Å	RMSD = 12.25 Å	RMSD = 16.29 Å	RMSD = 11.75 Å	RMSD = 11.65 Å
P-score = 0.02	_P-score_ = 0.05	_P-score_ = 0.01	_P-score_ = 0.02	_P-score_ = 0.01

Supplementary (3b)*, *Lig-01 (7JIV) *interaction energy (ΔE^{FMO}) (Kcal/mol) were calculated and shown on heatmap from Apo-opened P-loop SuMD simulations, against the binding site residues, for ligand Lig-01 PDB ID; 7JIV. RMSD of each pose was calculated against the heavy atoms of reference (crystal structure). P-score represents the prioritized pose. (No pose was prioritized as P-score was very low less than 0.10)*

Holo-closed **P-Loop conformation system -** ***Lig-02 (7JRN)***

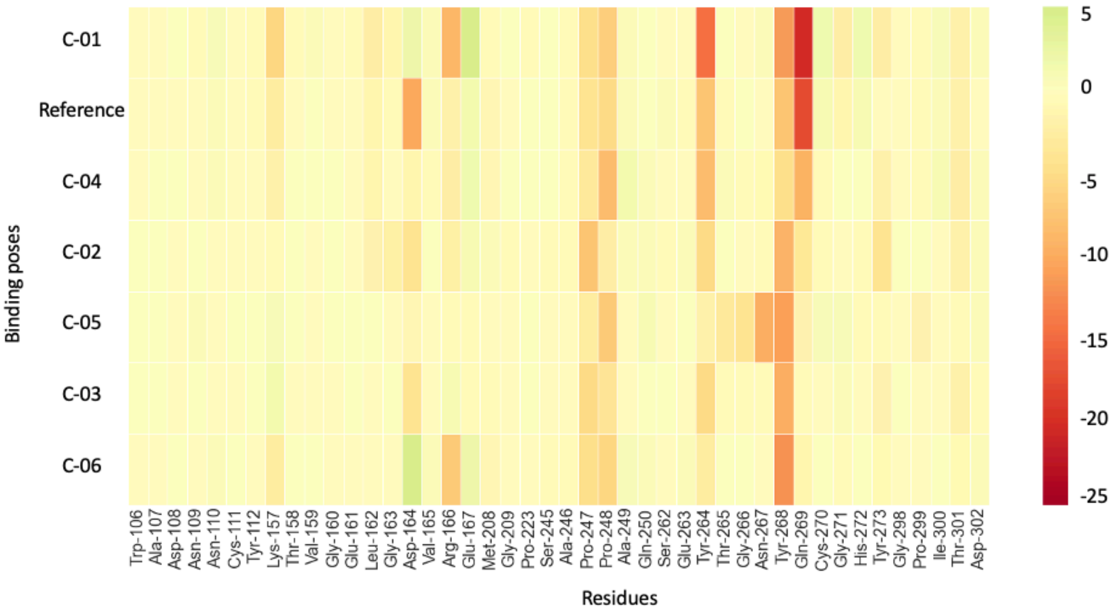

C0-1

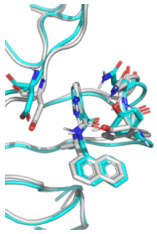

RMSD = 0.70 Å

P-score = 0.24

C0-2

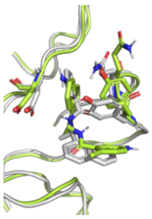

RMSD = 6.85 Å

P-score = 0.08

C0-3

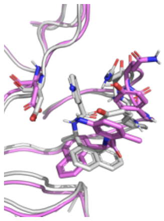

RMSD = 5.37 Å

P-score = 0.01

C0-4

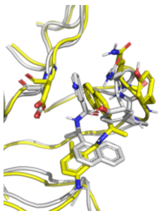

RMSD = 6.60 Å

P-score = 0.03

C0-5

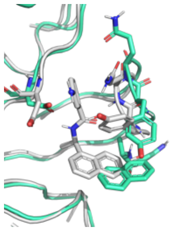

RMSD = 8.74 Å

P-score = 0.01

C0-6

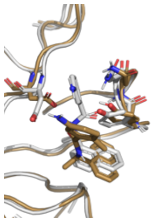

RMSD = 5.10 Å

P-score = 0.01

Supplementary (4a), ***Lig-02 (7JRN)*** *interaction energy (ΔE^{FMO}) (Kcal/mol) were calculated and shown on heatmap from Holo-closed P-loop SuMD simulations, against the binding site residues, for ligand Lig-02 PDB ID; 7JRN. RMSD of each pose was calculated against the heavy atoms of reference (crystal structure). P-score represents the prioritized pose.*

Apo-opened* P-Loop conformation system - *Lig-02 (7JRN)

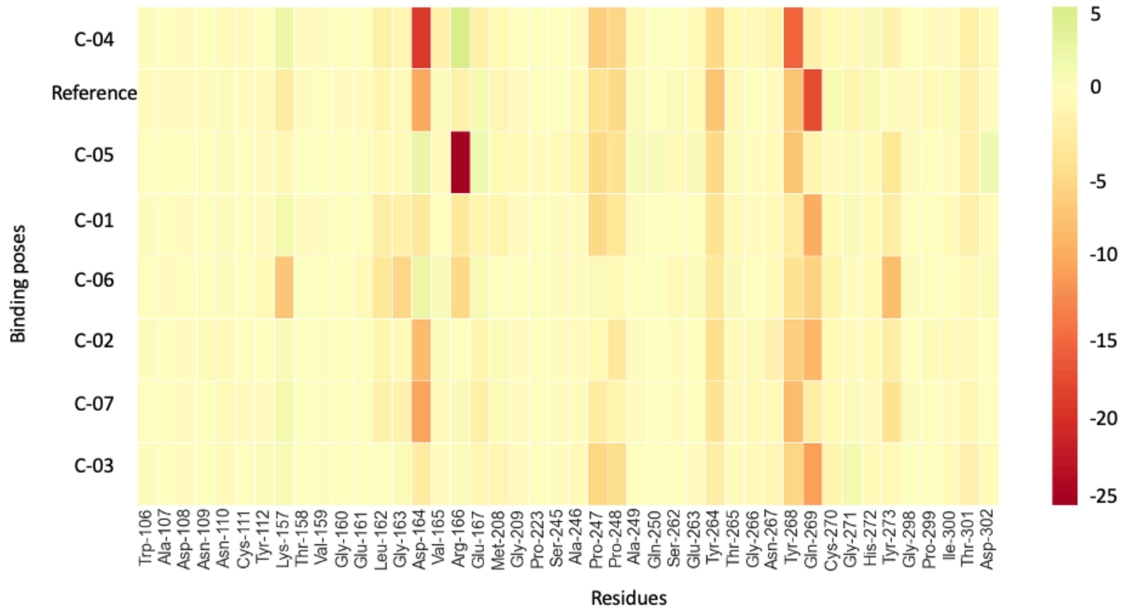

C0-1	C0-2	C0-3	C0-4	C0-5	C0-6	C0-7
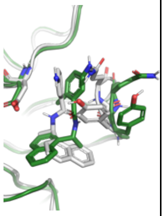	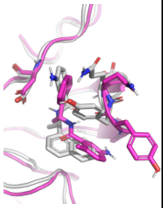	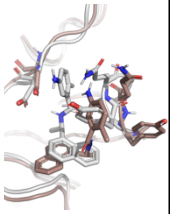	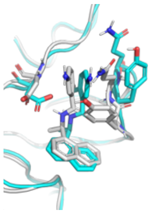	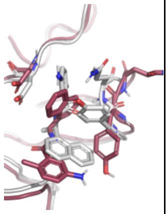	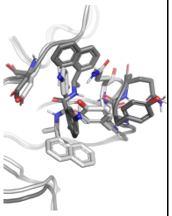	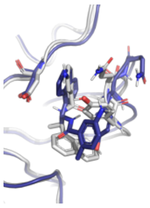
RMSD = 3.45 Å	RMSD = 7.01 Å	RMSD = 4.96 Å	RMSD = 1.32 Å	RMSD = 7.16 Å	RMSD = 7.51 Å	RMSD = 6.52 Å
P-score = 0.09	*P-score* = 0.06	*P-score* = 0.03	*P-score* = 0.10	*P-score* = 0.04	*P-score* = 0.02	*P-score* = 0.01

Supplementary (4b)*, *Lig-02 (7JRN) *interaction energy (ΔE^{FMO}) (Kcal/mol) were calculated and shown on heatmap from Apo-opened P-loop SuMD simulations, against the binding site residues, for ligand Lig-02 PDB ID; 7JRN. RMSD of each pose was calculated against the heavy atoms of reference (crystal structure). P-score represents the prioritized pose.*

Holo-closed* P-Loop conformation system - *Lig-03 (7RZC)

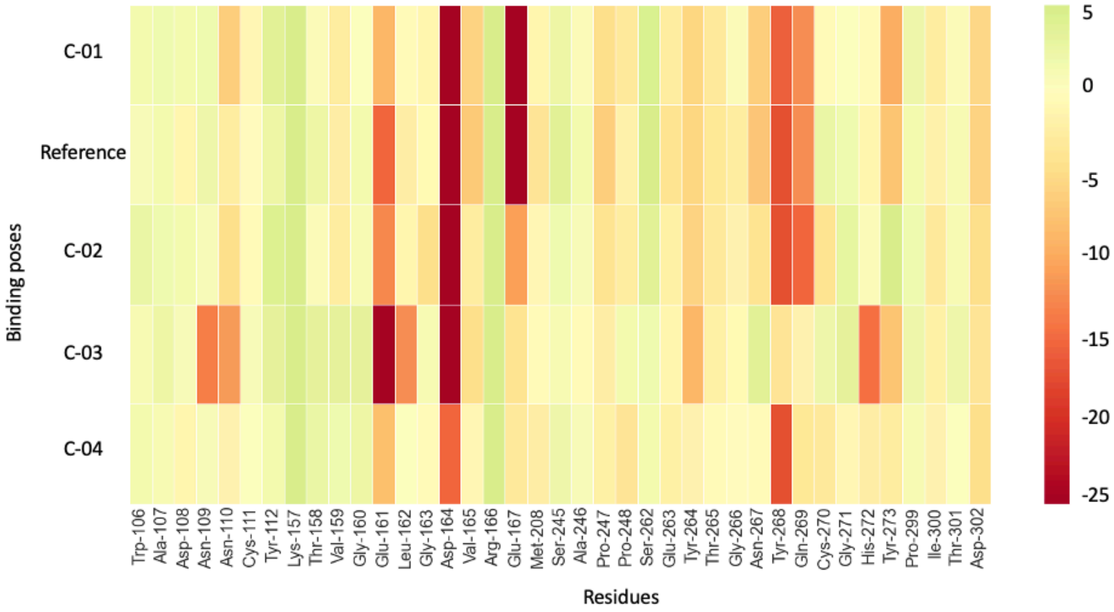

C0-1	C0-2	C0-3	C0-4
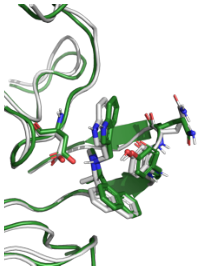	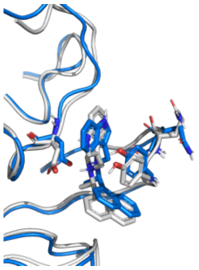	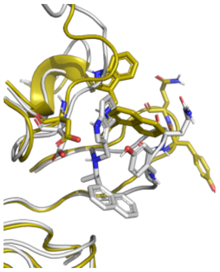	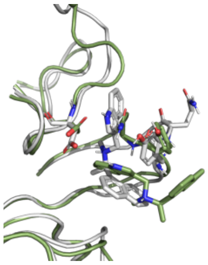
RMSD = 0.70 Å	RMSD = 2.10 Å	RMSD = 6.50 Å	RMSD = 6.40 Å
P-score = 0.31	*P-score* = 0.06	*P-score* = 0.02	*P-score* = 0.01

Supplementary (5a), Lig-03 (7RZC) *interaction energy (ΔE^{FMO}) (Kcal/mol) were calculated and shown on heatmap from Holo-closed P-loop SuMD simulations, against the binding site residues, for ligand Lig-03 PDB ID; 7RZC. RMSD of each pose was calculated against the heavy atoms of reference (crystal structure). P-score represents the prioritized pose.*

Apo-opened P-Loop conformation system - *Lig-03 (7RZC)*							
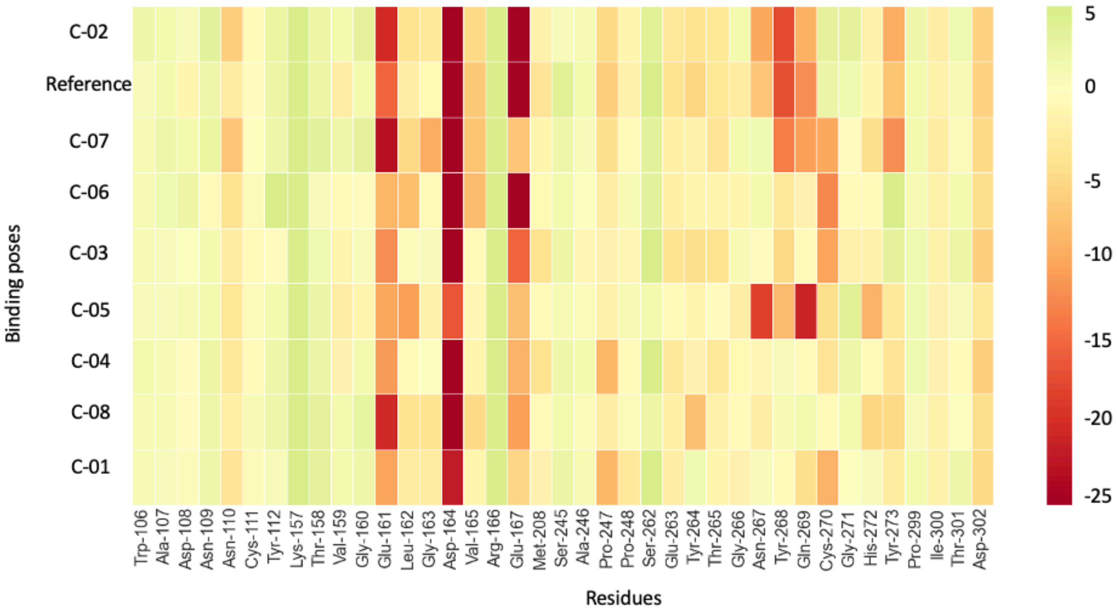							
C0-1	C0-2	C0-3	C0-4	C0-5	C0-6	C0-7	C0-8
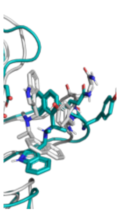	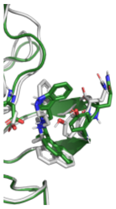	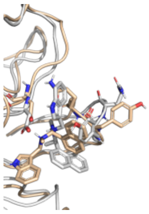	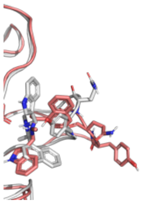	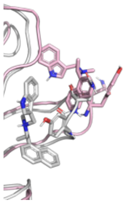	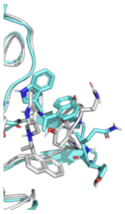	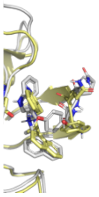	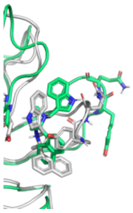
RMSD = 7.59 Å	RMSD = 1.69 Å	RMSD = 6.65 Å	RMSD = 7.07 Å	RMSD = 9.51 Å	RMSD = 6.75 Å	RMSD = 2.18 Å	RMSD = 3.97 Å
P-score = 0.01	*P-score =* 0.10	*P-score =* 0.04	*P-score =* 0.01	*P-score =* 0.01	*P-score =* 0.02	*P-score* = 0.02	*P-score =* 0.01

Supplementary (5b), ***Lig-03 (7RZC)*** *interaction energy (ΔE^{FMO}) (Kcal/mol) were calculated and shown on heatmap from Apo-opened P-loop SuMD simulations, against the binding site residues, for ligand Lig-03 PDB ID; 7RZC. RMSD of each pose was calculated against the heavy atoms of reference (crystal structure). P-score represents the prioritized pose.*

Holo-closed **P-Loop conformation system -** ***Lig-04 (7SDR)***

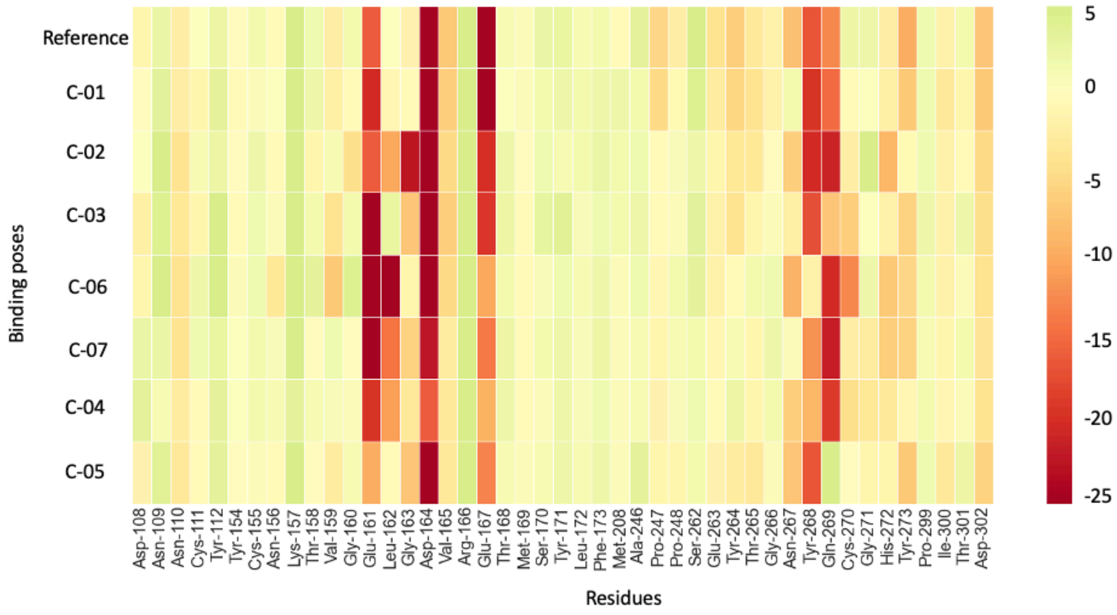

C0-1	C0-2	C0-3	C0-4	C0-5	C0-6	C0-7
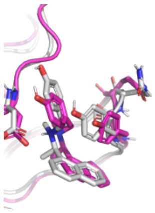	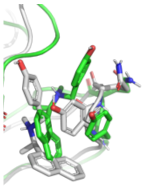	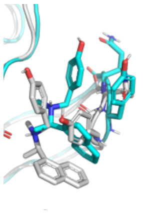	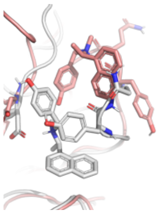	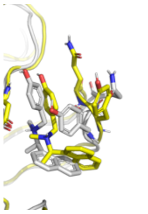	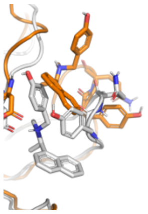	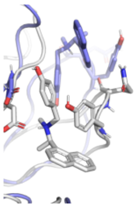
RMSD = 1.60 Å	RMSD = 3.90 Å	RMSD = 3.80 Å	RMSD = 7.60 Å	RMSD = 2.70 Å	RMSD = 6.80 Å	RMSD = 8.50 Å
P-score = 0.32	*P-score* = 0.08	*P-score* = 0.02	*P-score* = 0.01	*P-score* = 0.01	*P-score* = 0.01	*P-score* = 0.01

Supplementary (6a), ***Lig-04 (7SDR)*** *interaction energy (ΔE^{FMO}) (Kcal/mol) were calculated and shown on heatmap from Holo-closed P-loop SuMD simulations, against the binding site residues, for ligand Lig-04 PDB ID; 7SDR. RMSD of each pose was calculated against the heavy atoms of reference (crystal structure). P-score represents the prioritized pose.*

Apo-opened* P-Loop conformation system - *Lig-04 (7SDR)

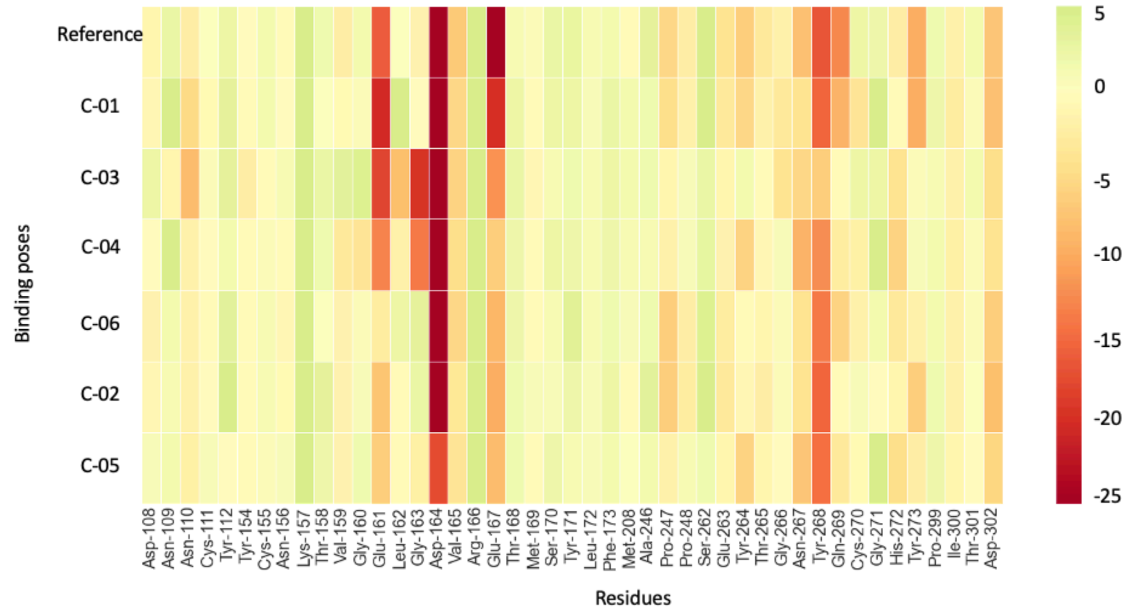

C0-1	C0-2	C0-3	C0-4	C0-5	C0-6
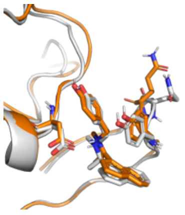	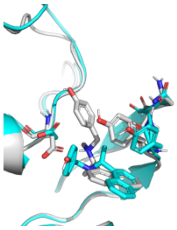	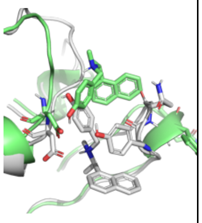	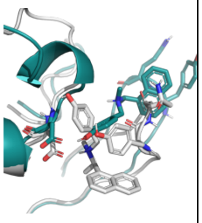	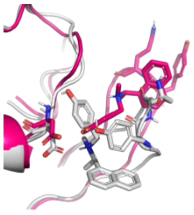	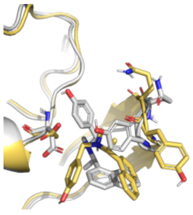
RMSD = 1.33 Å	RMSD = 4.57 Å	RMSD = 8.82 Å	RMSD = 8.24 Å	RMSD = 7.79 Å	RMSD = 5.06 Å
P-score = 0.57	*P-score* = 0.01	*P-score* = 0.02	*P-score* = 0.02	*P-score* = 0.01	*P-score* = 0.01

Supplementary (6b)*, *Lig-04 (7SDR) *interaction energy (ΔE^{FMO}) (Kcal/mol) were calculated and shown on heatmap from Apo-opened P-loop SuMD simulations, against the binding site residues, for ligand Lig-04 PDB ID; 7SDR. RMSD of each pose was calculated against the heavy atoms of reference (crystal structure). P-score represents the prioritized pose.*

Holo-closed* P-Loop conformation system - *Lig-05 (7TZJ)

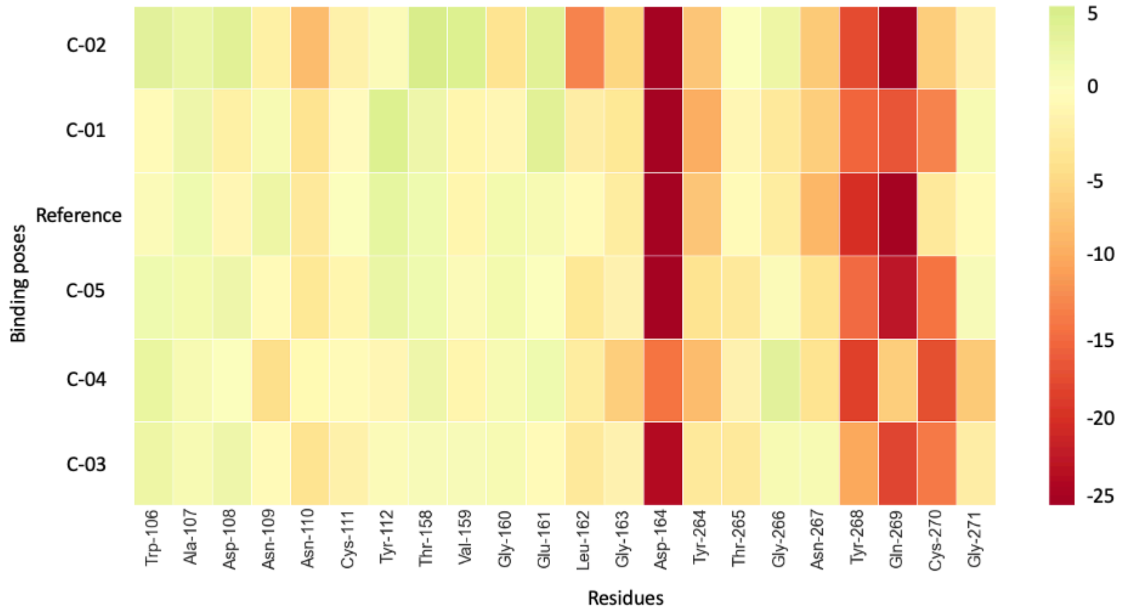

C0-1	C0-2	C0-3	C0-4	C0-5
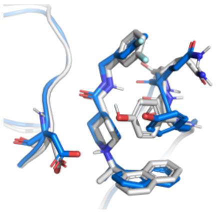	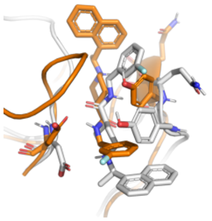	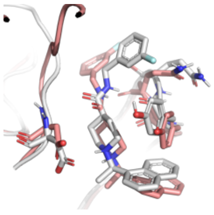	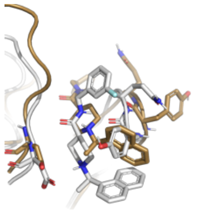	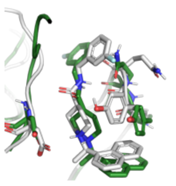
RMSD = 0.98 Å	RMSD = 10.41 Å	RMSD = 3.14 Å	RMSD = 6.23 Å	RMSD = 3.25 Å
P-score = 0.40	*P-score* = 0.09	*P-score* = 0.02	*P-score* = 0.01	*P-score* = 0.01

Supplementary (7a)*, *Lig-05 (7TZJ) *interaction energy (ΔE^{FMO}) (Kcal/mol) were calculated and shown on heatmap from Holo-closed P-loop SuMD simulations, against the binding site residues, for ligand Lig-05 PDB ID; 7TZJ. RMSD of each pose was calculated against the heavy atoms of reference (crystal structure). P-score represents the prioritized pose.*

Apo-opened* P-Loop conformation system - *Lig-05 (7TZJ)

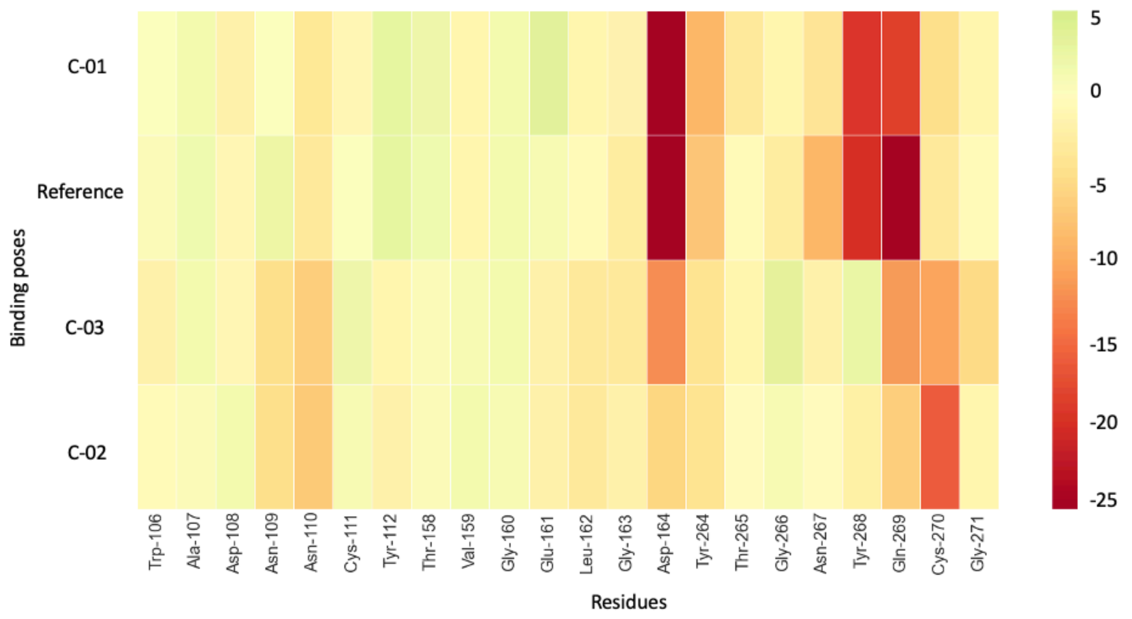

C0-1

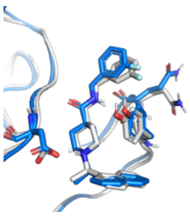

RMSD = 0.82 Å

P-score = 0.63

C0-2

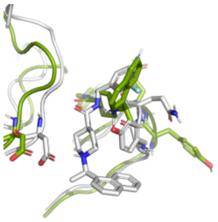

RMSD = 9.52 Å

P-score = 0.01

C0-3

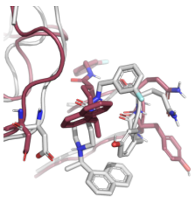

RMSD = 3.25 Å

P-score = 0.01

Supplementary (7b), Lig-05 (7TZJ) *interaction energy (ΔE^{FMO}) (Kcal/mol) were calculated and shown on heatmap from Apo-opened P-loop SuMD simulations, against the binding site residues, for ligand Lig-05 PDB ID; 7TZJ. RMSD of each pose was calculated against the heavy atoms of reference (crystal structure). P-score represents the prioritized pose.*

<table>
<tr><th>Ligands</th><th>Holo-closed P-Loop conformation system</th><th>Apo-opened P-Loop conformation system</th></tr>
<tr><td>Lig-01 (7JIV)</td><td>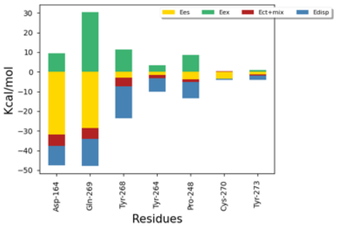
</td><td>NA</td></tr>
<tr><td>Lig-02 (7JRN)</td><td>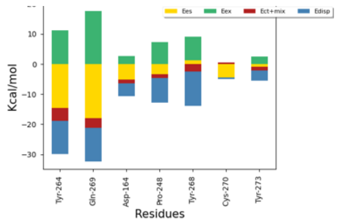
</td><td>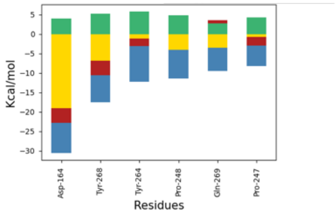
</td></tr>
<tr><td>Lig-03 (7RZC)</td><td>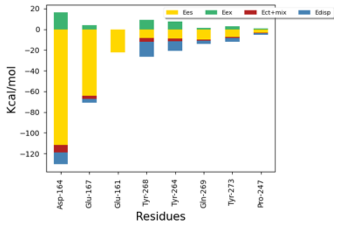
</td><td>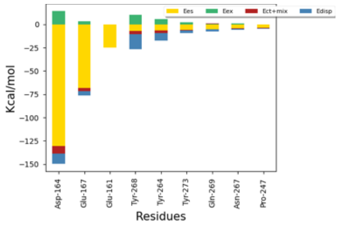
</td></tr>
<tr><td>Lig-04 (7SDR)</td><td>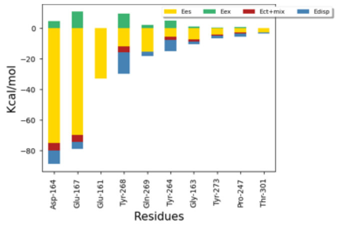
</td><td>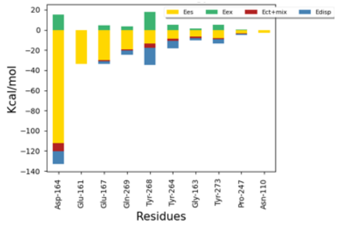
</td></tr>
<tr><td>Lig-05 (7TZJ)</td><td>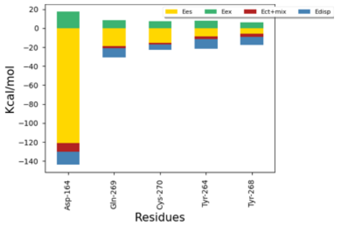
</td><td>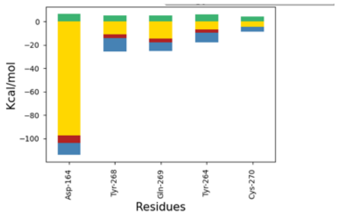
</td></tr>
<tr><td colspan="3">Supplementary (8), interaction energy (ΔE^{FMO}) (Kcal/mol) were calculated and shown on bar plots for selected pose with the highest P-score value from Holo-closed and Apo-open P-loop SuMD simulations, against the binding site residues, for each ligand (A) Lig-01 PDB ID; 7JIV, (B) Lig-02 PDB ID: 7JRN, (C) Lig-03 PDB ID: 7RZC, (D) Lig-04 PDB ID: 7SDR, and (E) Lig-05 PDB ID: 7TZJ.</td></tr>
</table>
