# Supplementary material for: Accurate prediction of dynamic protein–ligand binding using P‐score ranking
Source: J Comput Chem. 2024 Apr 22;45(20):1762–78. doi: 10.1002/jcc.27370 (PMC11980828; doi:10.1002/jcc.27370)
Supplement: Supplementary file 1 — Appendix S1: Supplementary Information. [file JCC-45-1762-s001.zip › Videos.pptx]

## Slide 1
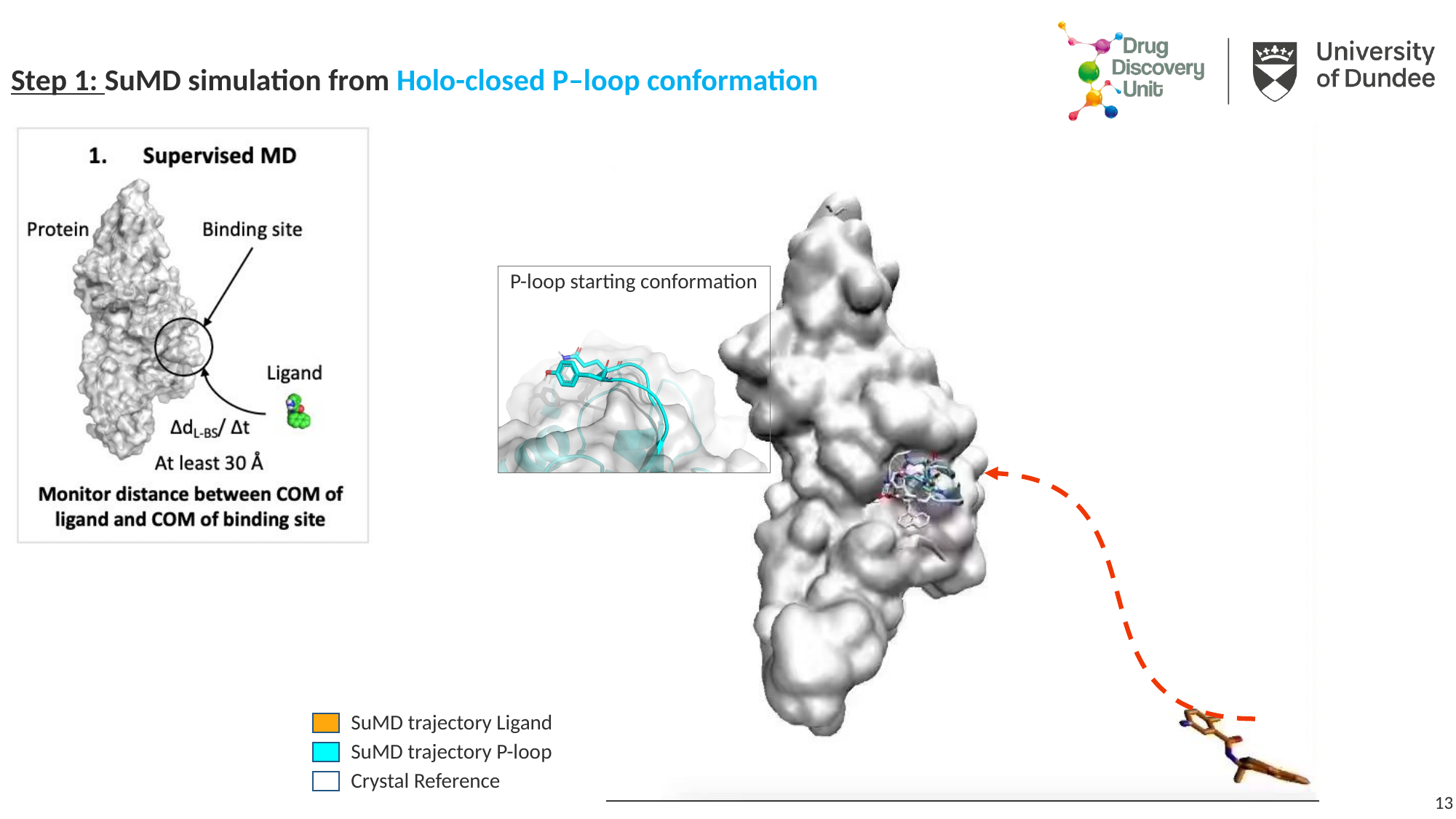

# Step 1: SuMD simulation from Holo-closed P–loop conformation
P-loop starting conformation
SuMD trajectory Ligand
SuMD trajectory P-loop
Crystal Reference
13

## Slide 2
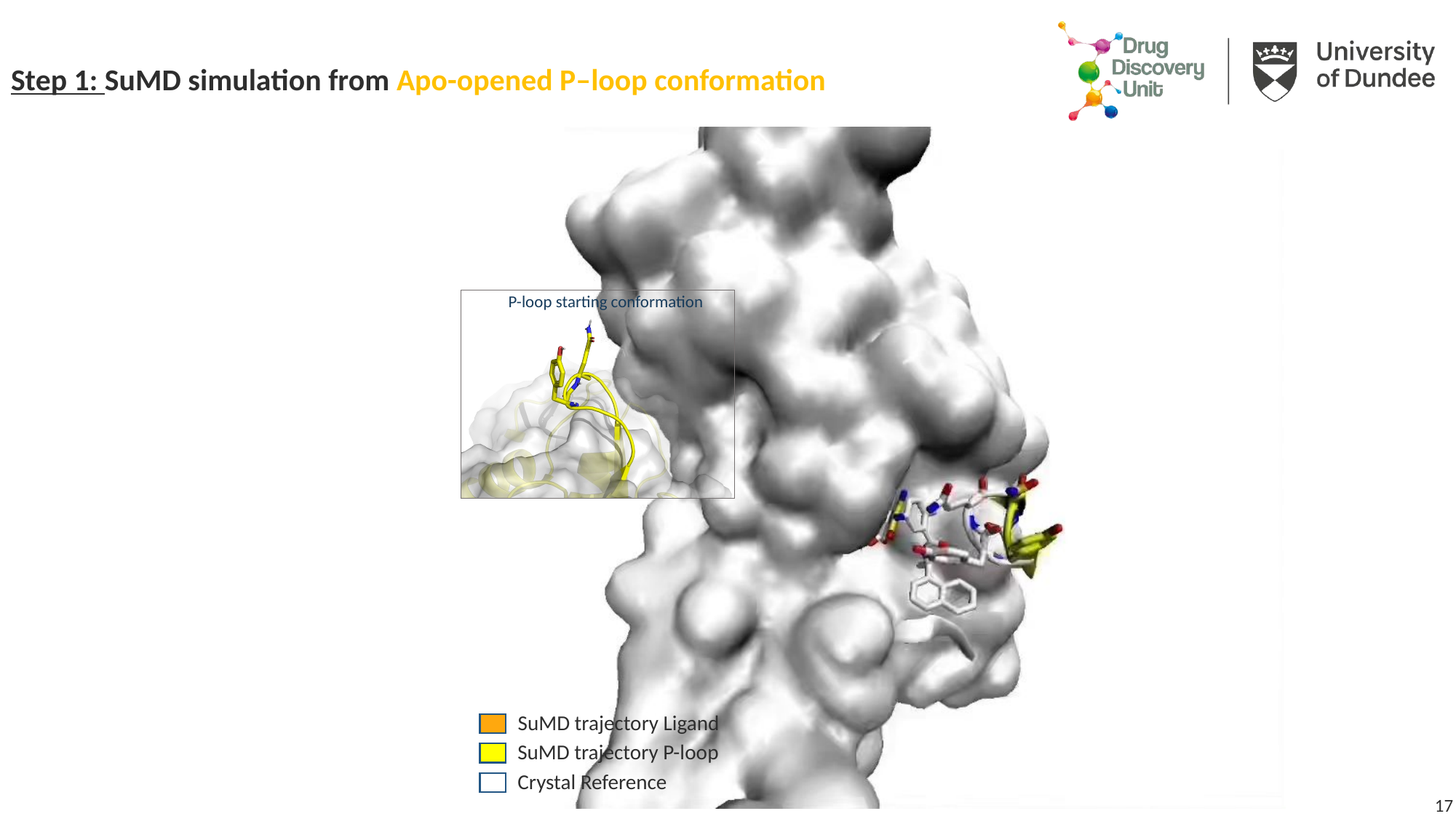

# Step 1: SuMD simulation from Apo-opened P–loop conformation
P-loop starting conformation
SuMD trajectory Ligand
SuMD trajectory P-loop
Crystal Reference
17

## Slide 3
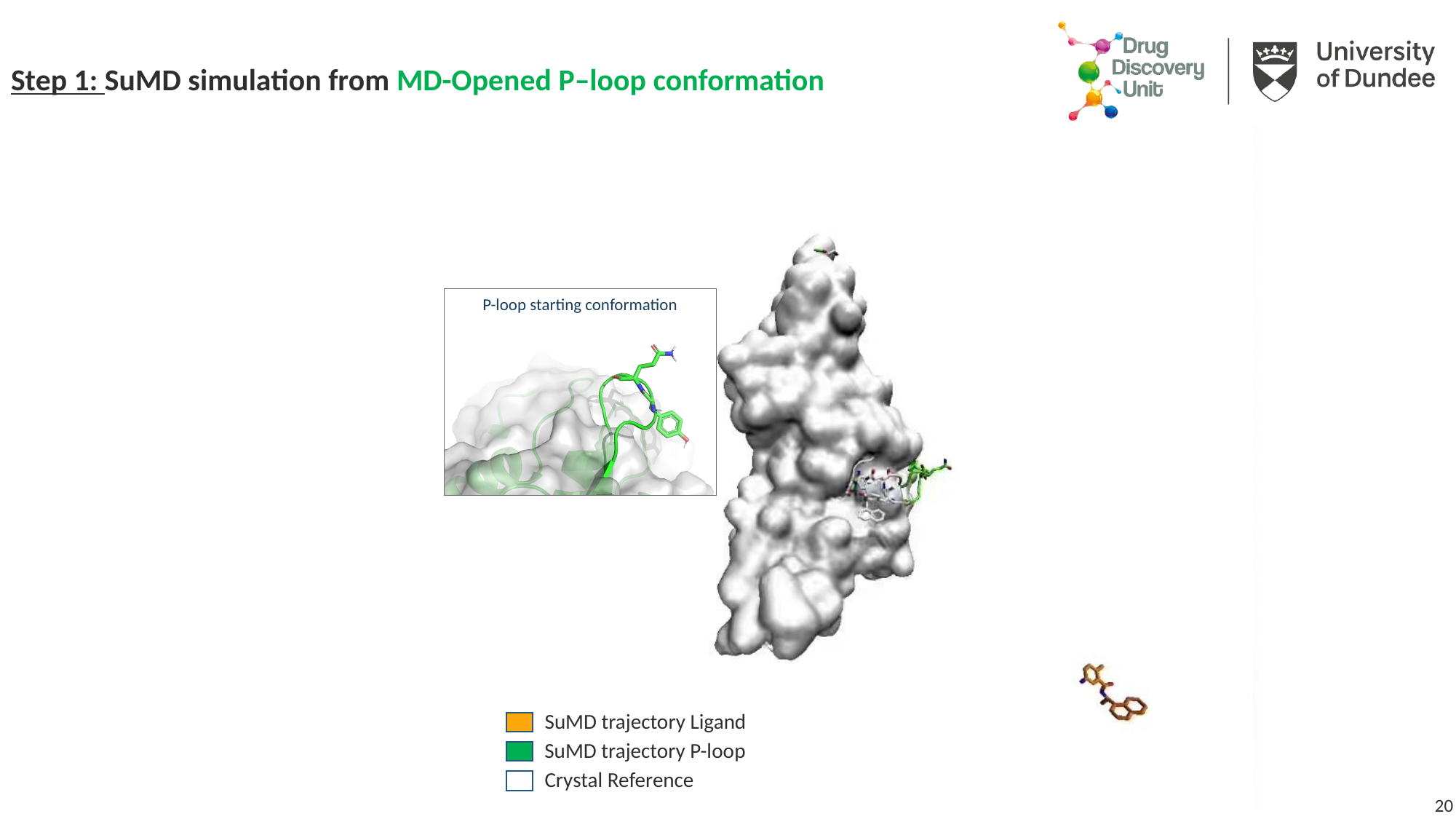

# Step 1: SuMD simulation from MD-Opened P–loop conformation
P-loop starting conformation
SuMD trajectory Ligand
SuMD trajectory P-loop
Crystal Reference
20
